# Molecular Basis of Acute Cystitis Reveals Susceptibility Genes and Immunotherapeutic Targets

**DOI:** 10.1371/journal.ppat.1005848

**Published:** 2016-10-12

**Authors:** Ines Ambite, Manoj Puthia, Karoly Nagy, Caterina Cafaro, Aftab Nadeem, Daniel S. C. Butler, Gustav Rydström, Nina A. Filenko, Björn Wullt, Thomas Miethke, Catharina Svanborg

**Affiliations:** 1 Division of Microbiology, Immunology and Glycobiology, Department of Laboratory Medicine, Lund University, Lund, Sweden; 2 Institute of Medical Microbiology and Hygiene, Medical Faculty of Mannheim, University of Heidelberg, Mannheim, Germany; University of Toronto, CANADA

## Abstract

Tissue damage is usually regarded as a necessary price to pay for successful elimination of pathogens by the innate immune defense. Yet, it is possible to distinguish protective from destructive effects of innate immune activation and selectively attenuate molecular nodes that create pathology. Here, we identify acute cystitis as an Interleukin-1 beta (IL-1β)-driven, hyper-inflammatory condition of the infected urinary bladder and IL-1 receptor blockade as a novel therapeutic strategy. Disease severity was controlled by the mechanism of IL-1β processing and mice with intact inflammasome function developed a moderate, self-limiting form of cystitis. The most severe form of acute cystitis was detected in mice lacking the inflammasome constituents ASC or NLRP-3. IL-1β processing was hyperactive in these mice, due to a new, non-canonical mechanism involving the matrix metalloproteinase 7- (MMP-7). ASC and NLRP-3 served as transcriptional repressors of *MMP7* and as a result, *Mmp7* was markedly overexpressed in the bladder epithelium of *Asc* ^*-/-*^ and *Nlrp3* ^*-/-*^ mice. The resulting IL-1β hyper-activation loop included a large number of IL-1β-dependent pro-inflammatory genes and the IL-1 receptor antagonist Anakinra inhibited their expression and rescued susceptible *Asc* ^*-/-*^ mice from bladder pathology. An MMP inhibitor had a similar therapeutic effect. Finally, elevated levels of IL-1β and MMP-7 were detected in patients with acute cystitis, suggesting a potential role as biomarkers and immunotherapeutic targets. The results reproduce important aspects of human acute cystitis in the murine model and provide a comprehensive molecular framework for the pathogenesis and immunotherapy of acute cystitis, one of the most common infections in man.

**Trial Registration:**

The clinical studies were approved by the Human Ethics Committee at Lund University (approval numbers LU106-02, LU236-99 and Clinical Trial Registration RTP-A2003, International Committee of Medical Journal Editors, www.clinicaltrials.gov).

## Author Summary

Infections continue to threaten human health as pathogenic organisms outsmart available therapies with remarkable genetic versatility. Fortunately, microbial versatility is matched by the flexibility of the host immune system which provide a rich source of novel therapeutic concepts. Emerging therapeutic solutions include substances that strengthen the immune system rather than killing the bacteria directly. Selectivity is a concern, however, as boosting of the antibacterial immune response may cause collateral tissue damage. This study addresses how the host response to urinary bladder infection causes acute cystitis and how this response can be attenuated in patients who suffer from this very common condition. We identify the cytokine Interleukin-1 beta (IL-1β) as a key immune response determinant in acute cystitis and successfully treat mice with severe acute cystitis by inhibiting IL-1β or the enzyme MMP-7 that processes IL-1β to its active form. Finally, we detect elevated levels of these molecules in urine samples from patients with cystitis, suggesting clinical relevance and a potential role of IL-1β and MMP-7 both as therapeutic targets and as biomarkers of infection. These findings provide a much needed, molecular framework for the pathogenesis and treatment of acute cystitis.

## Introduction

Acute cystitis is rapidly becoming a therapeutic enigma, as antibiotic resistance is reducing the options to a minimum [1–4]. Fortunately, new insights are now making it possible to explore immune response modifiers as alternatives to antibiotics. Acute cystitis occurs predominantly in girls and women with normal urinary tracts and at least 60% of all females will report an episode during their lifetime [5–7]. The recurrence rate is high, especially in a subset of patients, where severe, often recurrent cystitis episodes may cause chronic tissue damage and negatively impact the quality of life [8]. In addition, acute cystitis patients pose a highly significant challenge to the health care system. This study addresses if immunotherapy might be a relevant complement to antibiotics, in this patient group.

The urinary bladder mucosa is often exposed to bacteria but does not always retaliate with full force. In patients with acute cystitis, infection triggers a rapid and potent innate immune and inflammatory response in the bladder mucosa and clinical symptoms include pain, urgency and frequency of urination [9–12]. The molecular basis of these symptoms is not well understood, but bacterial interactions with the bladder epithelium have been shown to create inflammatory cascades [13–15], which also involve adjacent mucosal cells, such as mast cells and macrophages [16–20]. In asymptomatic carriers, the mucosa is exposed to bacteria of lower virulence and the mucosa remains fairly unresponsive, despite the presence of large numbers of bacteria in the lumen [21–24]. Asymptomatic bacteriuria (ABU) strains have evolved a mechanism to avoid elimination by the innate immune defense, through effects on RNA polymerase II and inhibition of host gene expression [22, 25]. It is therefore challenging to understand, at the molecular level, how a state of exaggerated mucosal inflammation can be generated specifically in acute cystitis patients. The specific molecular interactions that drive the transition from a homeostatic innate immune response to bladder disease remain unclear.

This study examined how innate immune response genes influence the outcome of bladder infection and the pathogenesis of acute cystitis. We identify acute cystitis as an IL-1β-driven, hyper-inflammatory disease [26, 27], possibly related to other hyper-inflammatory disorders [28, 29]. Consistent with such a role, *Il1b* ^*-/-*^ mice were protected from infection and pathology. In contrast *Asc* ^*-/-*^ and *Nlrp3* ^*-/-*^ mice developed progressive IL-1β-driven bladder inflammation and severe pathology, caused by a new, non-canonical IL-1β processing mechanism, involving the metalloproteinase MMP-7. We also identified the inflammasome constituents ASC (Apoptosis-associated speck-like protein containing a CARD) and NLRP-3 (NACHT, LRR and PYD domains-containing protein 3) as negative regulators of *MMP7*, explaining why MMP-7 is overexpressed in the mucosa of *Asc* ^*-/-*^ and *Nlrp3* ^*-/-*^ mice and the resulting state of IL-1β hyper-activation. Using IL-1β and MMP-7 as targets for immunotherapy, we succeeded in protecting susceptible *Asc* ^*-/-*^ mice against acute cystitis, confirming the potential of immunotherapy for this indication.

## Results

### Acute cystitis strains elicit an IL-1β response in human bladder epithelial cells

**Fig 1:**
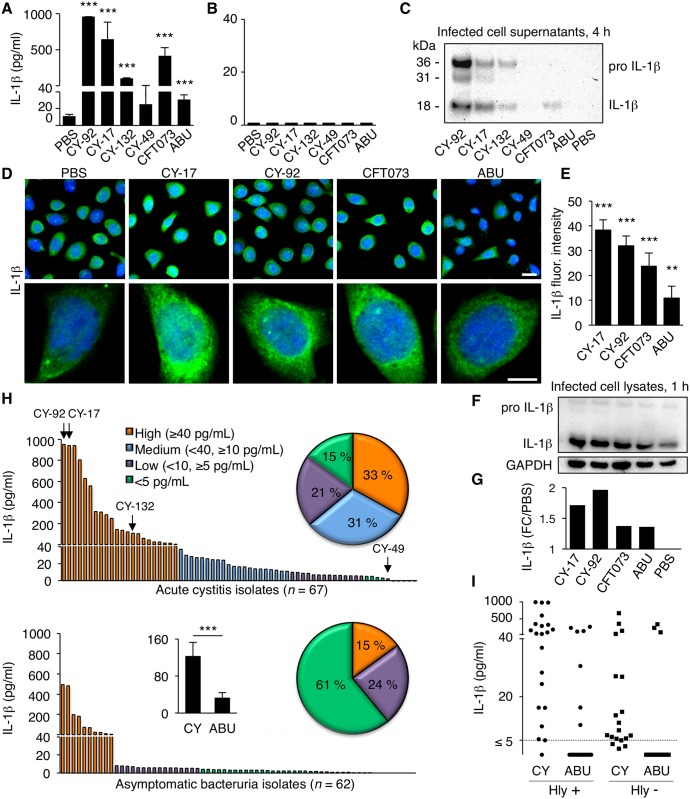
Acute cystitis strains activate an IL-1β response in human bladder epithelial cells. (**A**) IL-1β response in human bladder epithelial carcinoma cells (HTB-9) infected with acute cystitis strains CY-92, CY-17, CY-132 and CY-49 (4 hours). CFT073 and *E*. *coli* 83972 (ABU) were used as reference strains. CY-17, CY-92 and CY-132 triggered high IL-1β responses, as did CFT073. IL-1β was quantified, in cell supernatants, by ELISA (means ± SEMs of three independent experiments, *** *P* < 0.001 compared to PBS, two-tailed unpaired *t*-test). (**B**) Lack of IL-1β secretion by infected human kidney epithelial carcinoma cells (A498), infected as in **Fig 1A**. (**C**) Increased pro-IL-1β and mature IL-1β levels in HTB-9 cells infected with CY-92, CY-17 and CY-132 (Western blot analysis of cell supernatants, 4 hours, one representative experiment of several repeats). (**D**) IL-1β staining of HTB-9 cells infected with CY-17, CY-92, CY-132, CFT073 or ABU compared to the background in uninfected cells (PBS). Scale bars = 20 μm (upper panel) and 10 μm (lower panel). One representative experiment is shown. (**E**) Quantification of the cellular IL-1β response to infection. Increase in total fluorescence intensity (open pin-hole) after subtraction of the background staining in uninfected cells (PBS) (means ± SEMs of 50 cells per sample, ** *P* < 0.01 and *** *P* < 0.001 compared to PBS, two-tailed unpaired *t*-test). One of three experiments is shown. (**F, G**) IL-1β response to infection (1 hour) quantified by Western blot of whole cell extracts. Quantification of integrated density relative to GAPDH normalized against the background of uninfected cells. One representative experiment of several repeats. (**H**) IL-1β response to an epidemiologically defined collection of pediatric acute cystitis strains (*n* = 67) compared to ABU strains (*n* = 62), obtained from children in the same geographic area. IL-1β was quantified in infected cell supernatants, by ELISA. Pie chart depicting the frequency of bacterial strains activating IL-1β responses: high (orange), intermediate (blue), low (purple) or negative (green). Histogram (inset) of the mean IL-1β response to CY versus ABU strains (means ± SEMs, *** *P* < 0.001, two-tailed Mann Whitney test). (**I**) IL-1β activation plotted against hemolytic activity in the collection of CY and ABU strains. No significant association was detected (*n* = 18–21, Hly+ versus Hly-, two-tailed Mann Whitney test).

To address how infection creates a hyper-inflammatory state in patients with acute cystitis, we first infected the human bladder epithelial cell line HTB-9 *in vitro* and quantified inflammatory mediators in cell supernatants. We detected an increase in IL-1β secretion, four hours after infection with acute cystitis (CY) strains CY-17, CY-92, CY-132 or the uropathogenic *Escherichia coli* strain CFT073 (*P* < 0.001, compared to uninfected cells, two-tailed unpaired *t*-test). In contrast, the IL-1β response was low in cells infected with the ABU strain *E*. *coli* 83972, indicating a virulence-association (Fig 1A). IL-1β secretion was not detected in kidney epithelial cell supernatants after infection with the same strains, suggesting specificity for the bladder epithelium (Fig 1B). Western blot analysis confirmed that mature IL-1β was present in the supernatants of the infected HTB-9 cells (4 hours), as well as unprocessed pro-IL-1β and N-terminal fragment (Fig 1C). A rapid increase in IL-1β staining intensity was observed by confocal microscopy, in cells infected for one hour with 10^5^ CFU/ml of CY-17, CY-92 and CFT073 compared to uninfected cells or cells infected with the ABU strain (Fig 1D and 1E, two-tailed unpaired *t*-test). This increase in cellular IL-1β levels was confirmed by Western blot analysis of whole cell extracts (Fig 1F and 1G). At this time (1 hour), low levels of pro-IL-1β were detected. There was no significant reduction in cell viability after one (≥ 95% viable) or four hours (≥ 90% viable), as quantified by PrestoBlue staining and no evidence of pyroptosis after one hour, when the increase in cellular IL-1β levels was detected (S1 Fig). The presence of unprocessed pro-IL-1β in the 4 hour supernatant might be due to secretion of unprocessed IL-1β *via* exosomes [30].

To address if IL-1β activation is a characteristic of acute cystitis strains, we infected human bladder epithelial cells with an epidemiologically defined collection of pediatric acute cystitis isolates (*n* = 67, [31, 32]). The majority of these strains (85%) triggered an IL-1β response > 5 pg/ml and 64% of those triggered a high response (40–1,000 pg/ml, Fig 1H). We also examined a collection of pediatric ABU strains (*n* = 62, [31, 33]), which was obtained by screening infants and children in the same geographic area for bacteriuria in the absence of urinary tract infection (UTI) symptoms. In contrast to the CY strains, most of the ABU strains did not trigger a strong IL-1β response (61% < 5 pg/ml), resulting in significantly higher mean IL-1β concentrations in supernatants of bladder cells infected with the CY strains than the ABU strains (121.8 and 32.4 pg/ml respectively, *P* < 0.001, Fig 1H). To address if the secretion of IL-1β was influenced by bacterial hemolysin [19, 20], IL-1β concentrations were examined as a function of hemolytic activity in 40 CY and 38 ABU strains (Fig 1I). There was no significant difference in IL-1β response between hemolysin positive and negative strains (*P* = 0.07, Mann Whitney unpaired test). In the cystitis subset, a significant difference between hemolysin positive and negative strains was observed, however (*P* = 0.01, Mann Whitney unpaired test), suggesting that hemolytic cell lysis may contribute to the IL-1β activating virulent phenotype of the acute cystitis strains, for example by assisting the release of IL-1β from cells infected with hemolysin-producing strains.

The results suggest that the majority of acute cystitis strains activate an IL-1β response in human bladder epithelial cells.

### Genetic control of acute cystitis in the murine UTI model

**Fig 2:**
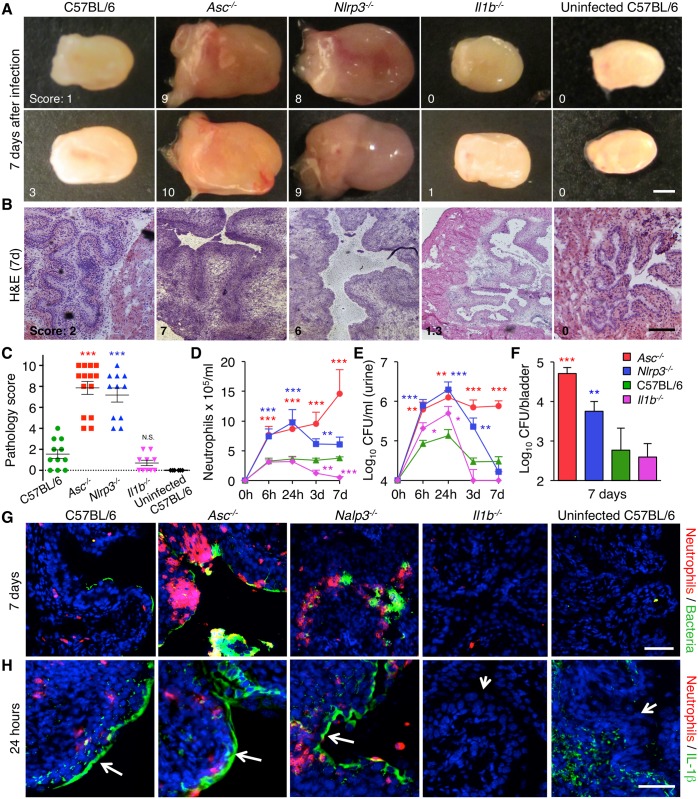
Genetic control of inflammation in acute cystitis. (**A**) Macroscopic evidence of acute cystitis in CFT073 infected mice, 7 days after infection. Slight increase in edema and hyperemia in C57BL/6 WT mice compared to uninfected controls, massively enlarged, hyper-inflamed bladders in *Asc* ^-/-^ and *Nlrp3* ^-/-^ mice and intact morphology in bladders from *Il1b* ^*-/-*^ mice. The macroscopic pathology score was based on edema, hyperemia and size on a scale of 0–10, where 10 the most edematous, hyperemic and largest bladder). Individual pathology scores are indicated. Scale bar = 1 mm. Two representative bladders are shown for each genotype. (For uninfected *Asc* ^-/-^, *Nlrp3* ^-/-^ and *Il1b* ^*-/-*^ mice see S2A Fig). (**B**) Bladder tissue structure in H&E-stained tissue sections. Bladders from *Asc* ^-/-^ and *Nlrp3* ^-/-^ mice showed extensive edema, a loss of tissue structure and epithelial hypertrophy, compared to C57BL/6 WT and *Il1b* ^*-/-*^ mice or uninfected control mice. The histology score was assessed using H&E stained bladder sections based on neutrophil infiltration, tissue architecture and epithelial thickness on a scale of 0–10, where 10 is highest neutrophil infiltration, least preserved tissue architecture and maximum epithelial thickness). Individual histology scores are indicated. Scale bar = 200 μm. (**C**) Gross bladder pathology score in infected mice (7 days) and uninfected C57BL/6 WT controls (means ± SEMs of two experiments, *** *P* < 0.001, two-tailed unpaired *t*-test compared to WT mice). (**D**) Elevated neutrophil counts and (**E**) bacterial counts in urine and (**F**) bladder tissues from *Asc* ^-/-^ and *Nlrp3* ^-/-^ mice, compared to WT and *Il1b* ^-/-^ mice. Means ± SEMs of two experiments, *** *P* < 0.001, ** *P* < 0.01, * *P* < 0.05, two-tailed unpaired *t*-test compared to WT mice. (**G**) Detection of neutrophils and bacteria in the mucosa of infected and control mice, by immunohistochemistry. Increased staining for mucosal neutrophils (red) and bacteria (green) in *Asc* ^-/-^ and *Nlrp3* ^-/-^ mice, compared to C57BL/6 WT, *Il1b* ^*-/-*^ mice and uninfected controls. Tissues obtained 7 days after infection. Scale bar = 50 μm. (**H**) Mucosal IL-1β staining in bladder tissue sections, obtained 24 hours after infection. Infection increased epithelial IL-1β staining in C57BL/6 WT, *Asc* ^-/-^ and *Nlrp3* ^-/-^ mice. Scale bar = 50 μm. Experiments included 14 *Asc* ^-/-^, 11 *Nlrp3* ^-/-^, 11 C57BL/6 WT and 10 *Il1b* ^-/-^ mice.

As IL-1β is processed by the inflammasome, we subsequently examined if mice with intact or defective inflammasome function develop acute cystitis. We infected mice with genetic defects affecting the NLRP-3 inflammasome: NLRP-3 deficient mice (*Nlrp3* ^-/-^ [34]) or ASC deficient mice (*Asc* ^-/-^ [35]). In addition, *Il1b* ^-/-^ [36] and *Casp1* ^-/-^ [37] mice were used and C57BL/6 WT mice were included as controls (Fig 2). For sample sizes and number of experiments, please see each figure legend and an overview in S1 Table. The mice were infected by intravesical inoculation with *E*. *coli* strains that triggered high IL-1β responses in human bladder epithelial cells, *in vitro* (CFT073, CY-17 or CY-92). Infected bladders were evaluated macroscopically, at sacrifice after 7 days and assigned a gross pathology score, defined by size, edema and hyperemia. Tissue pathology was further evaluated by hematoxylin and eosin (H&E) staining and immunohistochemistry of frozen tissue sections, and a histo-pathology score was assigned to each mouse. Histology was scored, independently, by two experienced researchers. The analysis was not blinded. Infection kinetics was followed in urine samples obtained after 6 and 24 hours, 3 and 7 days.

Major, genotype-specific differences in bladder inflammation and pathology were detected after seven days (Fig 2). Two disease end points were distinguished. 1. Severe, progressive cystitis in mice lacking ASC or NLRP-3, resembling chronic human disease. 2. A moderate, self-limiting form of acute cystitis in C57BL/6WT mice with intact inflammasome function, resembling sporadic human cystitis.

Infected *Asc* ^*-/-*^ and *Nlrp3* ^-/-^ mice developed severe, acute cystitis with enlarged bladders, edema and hyperemia compared to uninfected bladders (Fig 2A and S2A Fig). By histology, most bladders from *Asc* ^*-/-*^ mice showed extensive loss of tissue structure, defined by a pronounced mucosal and submucosal edema, with disappearance of the tissue folds that characterizes the healthy mucosa. In addition, a massive inflammatory cell infiltrate was present, along the mucosal border and in the submucosa (10/14 mice, 71%). Similar tissue destruction was observed in bladders from *Nlrp3* ^-/-^ mice (7/11 mice, 64%), (Fig 2B). Mean gross bladder pathology score of infected *Asc* ^*-/-*^ and *Nlrp3* ^-/-^ mice were 7.9 and 7.2, respectively (Fig 2C).

Bladder pathology was accompanied by high bacterial counts in urine and bladder tissue (Fig 2D–2F). In *Asc* ^-/-^ mice, the neutrophil influx accelerated until day 7, indicating a loss of homeostatic control and progression to chronic inflammation. Infiltrating bacteria and neutrophil aggregates or micro-abscesses were detected in the mucosa of *Asc* ^*-/-*^ and *Nlrp3* ^*-/-*^ mice, with extensive sloughing of epithelial cells into the lumen. Bacteria were mainly localized along the mucosal surface, with no evidence of bacterial invasion (Fig 2G). This hyper-inflammatory phenotype was also observed after infection of *Asc* ^-/-^ mice with the acute cystitis strains CY-92 and CY-17, which triggered high IL-1β responses *in vitro* (S3A–S3D Fig). In contrast, there was no disease phenotype in *Asc* ^-/-^ mice infected with the ABU strain *E*. *coli* 83972 or in C57BL/6 WT mice after 24 hours or 7 days (S3E Fig). There was no evidence of kidney involvement or pathology in mice infected with CY-92 and CY-17, despite positive bacterial cultures from renal tissues.

In contrast, C57BL/6 WT mice infected with CFT073 showed moderate macroscopic evidence of acute cystitis including a small increase in size, edema and hyperemia compared to uninfected controls (Fig 2A and 2C, mean pathology score 1.5). The low level of edema was confirmed by histology, with no evidence of tissue damage (Fig 2B). Infection was accompanied by an increase in urine neutrophil numbers (Fig 2D) and bacterial numbers reached a peak after 24 hours and then declined (Fig 2E and 2F). By immunohistochemistry, bacterial staining was weak and very few neutrophils were detected in the bladder mucosa (Fig 2G).

*Il1b* ^-/-^ mice showed an even more attenuated phenotype after infection with CFT073, consistent with a key role of IL-1β for bladder inflammation and pathology compared to uninfected bladders (Fig 2A and S2A Fig). There was no macroscopic evidence of acute cystitis (Fig 2A and 2C, mean pathology score 0.7) and bladder tissue morphology remained intact (Fig 2B, mean histo-pathology score 0.9, *P* = 0.003 compared to C57BL/6 WT mice). *Il1b* ^-/-^ mice had fewer infiltrating neutrophils and lower bacterial counts than the C57BL/6 WT mice on day seven (*P* < 0.001 and *P* < 0.05), (Fig 2D–2F). Furthermore, *Casp1* ^*-/-*^ mice, which have a functional IL-1β deficiency due to defective IL-1β processing and secretion [37], did not develop acute cystitis (S4A–S4D Fig). The bladders were enlarged and hyperemic, but there was no evidence of inflammatory changes or tissue damage. *Casp1* ^*-/-*^ mice showed reduced IL-1β secretion and tissue retention of IL-1β (S4E–S4G Fig). As a result, IL-1β dependent gene expression was low and *Casp1* ^*-/-*^ mice showed a lack of inflammation in bladder tissues. Neutrophils and bacteria were present in urine but did not accumulate in the tissues and the mucosal morphology was intact (S4 Fig).

Infection was accompanied by strong mucosal IL-1β staining in bladder tissue sections in C57BL/6 WT mice, *Asc* ^*-/-*^ and *Nlrp3* ^*-/-*^ mice after 24 hours (Fig 2H). Staining was mainly epithelial and was not seen in *Il1b* ^*-/-*^ mice or uninfected C57BL/6 WT mice. In parallel with the epithelial staining IL-1β was detected by ELISA in the urine of infected *Asc* ^*-/-*^ and *Nlrp3* ^*-/-*^ mice, with lower levels in C57BL/6 WT mice. By Western blot analysis, bands of approximately 36 and 18 kDa were detected (S2B Fig).

These studies identify genetic determinants of host susceptibility to acute cystitis. *Asc* and *Nlrp3* were defined as key resistance determinants and IL-1β activation as a crucial step in the pathogenesis of acute cystitis.

### *Asc* and *Nlrp3* control gene expression in infected bladders

To define the mechanism of bladder pathology, we extracted total bladder RNA from infected *Asc* ^*-/-*^ and *Nlrp3* ^*-/-*^ mice with the highest pathology score after seven days and from C57BL/6 WT and *Il1b* ^*-/-*^ mice, with low pathology scores (Experiments 1 and 2 in S1 Table) and from uninfected bladders. The RNA was amplified, hybridized onto Mouse Genome array strips, washed, stained and scanned using the GeneAtlas system. Significantly altered genes were identified, by comparing infected- to uninfected mice of the same genetic background (*P*-values < 0.05 and absolute fold change > 1.41) and sorted by relative expression using 2-way ANOVA [38]. Heat-maps were constructed by Gitools 2.1.1 software and differentially expressed genes and regulated pathways were analyzed by Ingenuity Pathway Analysis software (see Materials and Methods).

**Fig 3:**
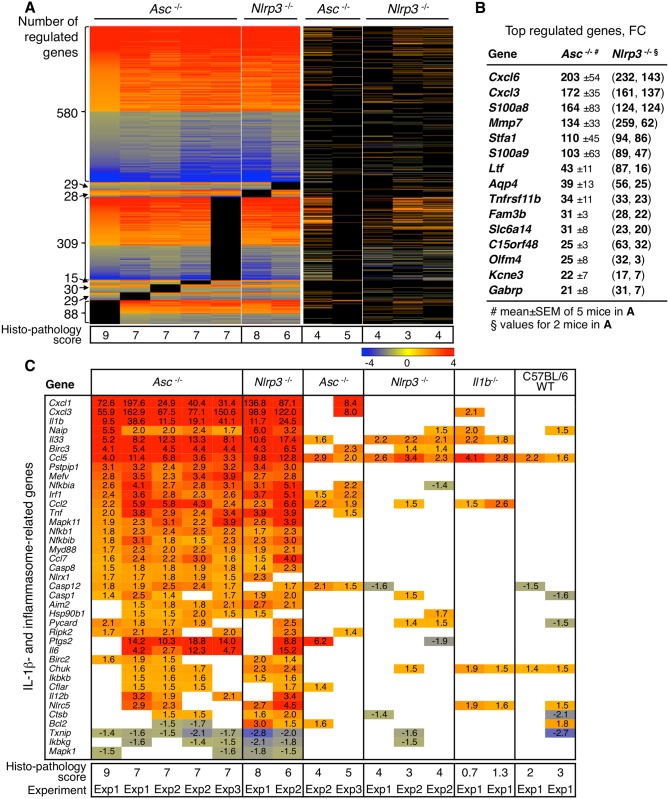
Hyper-activation of IL-1β dependent gene expression and bladder pathology in *Asc* ^*-/-*^ and *Nlrp3* ^*-/-*^ mice. Transcriptomic analysis of whole bladder RNA from infected mice (CFT073, 7 days), compared to uninfected controls of each genotype (cut off FC 1.41, *P* < 0.05). (**A**) Heatmap of regulated genes in *Asc* ^*-/-*^ and *Nlrp3* ^*-/-*^ mice with the highest bladder pathology score, defined by neutrophil infiltration, loss of tissue structure and epithelial thickness in H&E stained bladder tissue sections. Scale FC -4 to 4, red = upregulated, blue = downregulated. A distinct gene set distinguished the *Asc* ^*-/-*^ and *Nlrp3* ^*-/-*^ mice with a high histopathology score from C57BL/6 WT mice or *Il1b* ^*-/-*^ mice without pathology. (**B**) Top up-regulated genes in the pathology-associated gene set, compared to uninfected controls of each genotype. Means ± SEMs of 5 mice for *Asc* ^*-/-*^ mice and 2 *Nlrp3* ^*-/-*^ mice. (**C**) Analysis of IL-1β, inflammasome activators and effectors in *Asc* ^*-/-*^ and *Nlrp3* ^*-/-*^ mice, detecting massive over-expression compared to *Il1b* ^*-/-*^ and WT mice. Red = upregulated, blue = suppressed. The data set included gene expression profiles from 7 *Asc* ^*-/-*^ and 5 *Nlrp3* ^*-/-*^ mice, and two each of the C57BL/6 WT and *Il1b* ^*-/-*^ controls. Uninfected control RNA of each genotype were used to define significantly regulated genes (≥ 2 mice per genotype). Histopathology scores and group numbers for individual mice (see also Experiments 1, 2 and 3 in S1 Table).

We identified a set of strongly upregulated genes in *Asc* ^*-/-*^ and *Nlrp3* ^*-/-*^ mice with the highest bladder pathology score, but not in C57BL/6 WT mice or *Il1b* ^*-/-*^ mice (2,228 specifically regulated genes). The heat map in Fig 3A illustrates the similarities in gene expression between 5/7 *Asc* ^*-/-*^ and 2/5 *Nlrp3* ^*-/-*^ mice analyzed by this technology. Those mice also had high and comparable histology scores, defined by evaluation of the H&E-stained bladder tissue sections from the corresponding mice. To further understand the disease process, we identified the most strongly upregulated genes in these mice. Genes with a FC > 100 included metalloproteinase *Mmp7*, the neutrophil and monocyte chemoattractants *Cxcl6* and *Cxcl3*, the genes encoding calprotectin *S100a8* and *a9* and the stefin gene *Stfa1* (Fig 3B and S2 Table). By analysis of top-scoring canonical pathways, these genes were shown to control granulocyte and leucocyte diapedesis and signaling, acute phase responses including IL-6 and IL-1β signaling, IL-1R expression and NF-κB-signaling and dendritic cell maturation (S5A Fig). These genes and pathways were not significantly regulated in C57BL/6 WT or *Il1b* ^-/-^ mice, supporting a disease association.

The activation of inflammasome-genes and IL-1β-dependent gene network was analyzed, using Qiagen’s list of 84 key inflammasome genes (Fig 3C). In the *Asc* ^-/-^ and *Nlrp3* ^*-/-*^ mice with a high histo-pathology score, *Il1b* expression was activated (FC 10–41), as were IL-1β-dependent and inflammasome-related genes including *Cxcl1*, *Cxcl3* and *Il33* (FC 5–200), (Fig 3C). These genes and pathways were not significantly regulated in C57BL/6 WT or *Il1b* ^*-/-*^ mice (Fig 3C). *Il1a* expression was activated in *Asc* ^*-/-*^ and *Nlrp3* ^*-/-*^ mice with bladder pathology, but not among the 40 top-regulated genes (FC 1.6–8.7). *Il18*, *Casp11* and inflammasome-related NLRP genes were not transcriptionally regulated (S2 Table).

The results identify IL-1β driven pro-inflammatory genes that are activated, exclusively in *Asc* ^*-/-*^ and *Nlrp3* ^*-/-*^ mice with severe bladder pathology. This response was not detected in the kidneys of infected C57BL/6 WT mice (S5B Fig).

### Mechanism of atypical IL-1β processing in infected bladders

**Fig 4:**
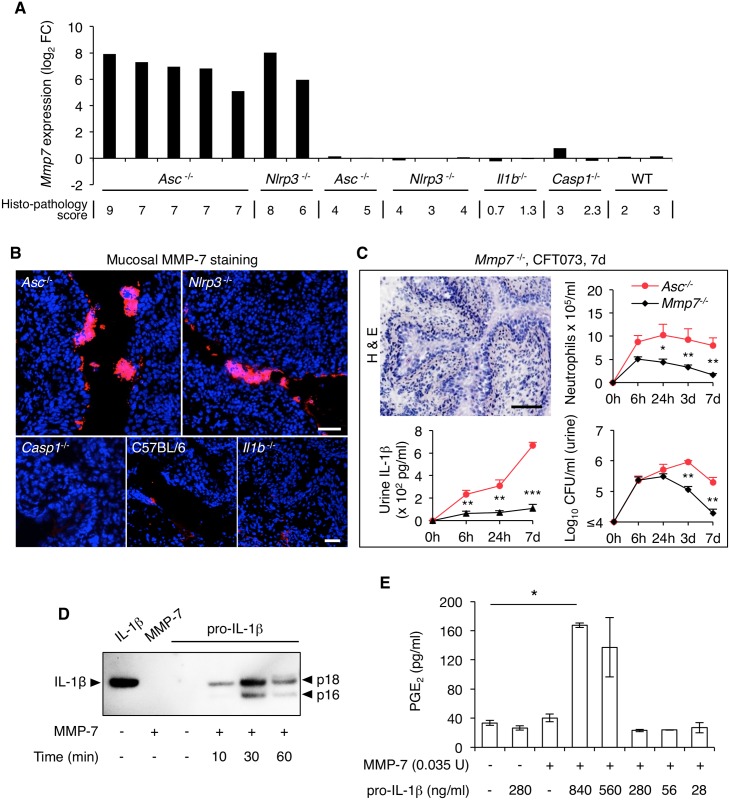
IL-1β processing by MMP-7. (**A**) Gene expression profiling identified *Mmp7* as one of the top up-regulated gene in *Asc* ^*-/-*^ and *Nlrp3* ^*-/-*^ mice with bladder pathology (CFT073 infected mice, 7 days). Log_2_ fold change of *Mmp7* expression levels in individual mice are shown relative to the H&E pathology score. *Mmp7* was not regulated in C57BL/6 WT mice or in *Il1b* ^*-/-*^ or *Casp1* ^*-/-*^ mice. (**B**) Strong epithelial MMP-7 staining in *Asc* ^*-/-*^ and *Nlrp3* ^*-/-*^ mice with bladder pathology. MMP-7 staining was very low in C57BL/6 WT, *Il1b* ^*-/-*^ and *Casp1* ^*-/-*^ mice. Scale bars = 50 μm. (**C**) Phenotype of *Mmp7* ^-/-^ mice, 7 days after infection with CFT073. Intact mucosal tissue structure with inflammatory cell infiltration. Low bacterial and neutrophil counts in urine compared to *Asc* ^-/-^ mice (*n* = 5 mice per group, means ± SEMs, ** *P* < 0.01, *** *P* < 0.001, two-tailed unpaired *t*-test). Scale bar = 1 mm. IL-1β levels were elevated in the urine of *Asc* ^-/-^ mice but not in *Mmp7* ^-/-^ mice, as detected by ELISA. (**D**) Proteolytic cleavage of pro-IL-1β by MMP-7 *in vitro*, using purified enzyme and GST-tagged pro-IL-1β. The IL-1β fragments generated by proteolysis were 18 and 16 kDa, defined by Western blot using an antibody specific for the mature form of IL-1β. Recombinant mature IL-1β and GST-tagged pro-IL-1β were used as controls, as well as recombinant MMP-7. One representative experiment out of three, see also S7A Fig. (**E**) Bioassay for IL-1β activity, measuring the PGE_2_ response of human bladder epithelial cells to the IL-1β fragments generated by MMP-7 proteolysis of GST-tagged pro-IL-1β. The cleaved products activated PGE_2_ but MMP-7 and pro-IL-1β had no effect (means ± SEMs of two experiments, ** *P* < 0.01, two-tailed Mann Whitney test).

The *Mmp7* gene, which encodes the matrix metalloproteinase (MMP)-7 [39] was strongly upregulated in *Asc* ^*-/-*^ and *Nlrp3* ^*-/-*^ mice with a high histo-pathology score (Fig 3B). MMP-7 expression was therefore examined as a function of the histo-pathology score (Fig 4A). In *Asc* ^*-/-*^ and *Nlrp3* ^*-/-*^ mice, *Mmp7* expression showed a clear association to the overall bladder tissue pathology score and was not regulated in the *Il1b* ^*-/-*^ or C57BL/6 WT mice (Fig 4A). High MMP-7 protein expression was confirmed, by immunohistochemistry, in bladder tissue sections from *Asc* ^*-/-*^ and *Nlrp3* ^*-/-*^ mice (Fig 4B). Importantly, staining was exclusively epithelial, with shedding of MMP-7 positive cells into the bladder lumen. Epithelial MMP-7 activation was detected as early as 24 hours after infection and importantly, MMP-7 showed no detectable co-localization with neutrophils in the mucosa or sub-mucosa (S6 Fig) To further evaluate the involvement of MMP-7 in acute cystitis, we infected *Mmp7* ^*-/-*^ mice [40] with CFT073 and used *Asc* ^-/-^ mice as disease controls. Consistent with their intact inflammasome function, *Mmp7* ^*-/-*^ mice developed transient cystitis similar to C57BL/6 WT mice (Fig 4C). A moderate mucosal IL-1β response was observed by immunohistochemistry (Fig 4C). IL-1β levels in urine (Fig 4C) and IL-1β-dependent gene expression was comparable to that in WT mice, with expression of *Ccl5*, *Nlrc5*, *Irf1*, *Ctsb*, *Birc3*, and *MyD88*. Thus, *Mmp7* did not drive pathology in mice with intact ASC or NLRP-3 function.

To address if MMP-7 cleaves IL-1β, we exposed recombinant GST-tagged pro-IL-1β to recombinant active MMP-7 *in vitro* and detected proteolytic fragments by Western blot, using IL-1β specific antibodies (Fig 4D and S7A Fig). Kinetic analysis detected a time-dependent cleavage of IL-1β with a reduction in full-length protein from 10 to 60 minutes (Fig 4D). Using antibodies with higher affinity for the mature IL-1β, a band of 18 kDa was detected corresponding in size to the recombinant, active and mature IL-1β control. With increasing time, a band of 16 kDa was also observed (Fig 4D). Using the same experimental set up, we observed that ASC was degraded by MMP-7 over time (S7B Fig) while recombinant NLRP-3 was not cleaved by MMP-7 and therefore served as a negative control for unspecific effects of the enzyme (S7C Fig).

To address if the cleaved IL-1β fragments were biologically active, reaction mixtures containing pro-IL-1β and MMP-7 were collected after 30 minutes, when the mature product was detected by Western blot (Fig 4D). Human bladder epithelial cells were stimulated with the reaction mixture for one hour, and IL-1β activity was quantified, by measuring the prostaglandin E2 (PGE_2_) response [41]. The 30 minutes reaction mixture activated a dose-dependent PGE_2_ response but recombinant MMP-7 and pro-IL-1β (280 and 840 ng/ml) alone had no effect (Fig 4E).

The results identify a new, MMP-7-dependent mechanism of pro-IL-1β processing in *Asc* ^-/-^ and *Nlrp3* ^*-/-*^ mice.

### NLRP-3 and ASC act as negative regulators of *MMP7* expression

**Fig 5:**
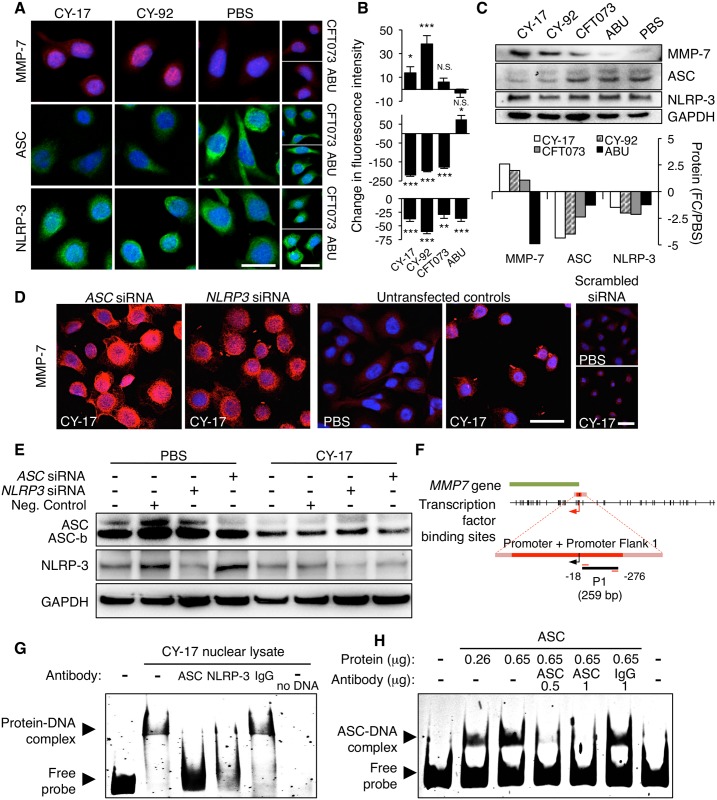
Regulation of *MMP7* expression by ASC and NLRP-3. (**A**) MMP-7, ASC and NLRP-3 responses to infection were visualized by confocal microscopy. An increase in MMP-7 and decrease in ASC staining were detected after infection of HTB-9 cells with CY-17 and CY-92 for 1 hour, compared to uninfected control cells. NLRP-3 staining was weakly affected. (**B**) Quantification of total fluorescence intensity (open pin-hole) after subtraction of the background staining in uninfected cells (PBS). Medians ± SEMs of 50 cells, * *P* < 0.05, ** *P* < 0.01, *** *P* < 0.001, compared to PBS control, two-tailed unpaired *t*-test (MMP-7 and NLRP-3) or two-tailed Mann Whitney test (ASC). One of three experiments is shown. (**C**) Western blot confirming the change in cellular content of MMP-7, ASC and NLRP-3, 1 hour after infection with the indicated strains. Fold change compared to PBS of normalized values (against GAPDH). One experiment out of 2 is shown. (**D**) Increase in MMP-7 expression in HTB-9 cells transfected with siRNAs specific for *ASC* or *NLRP3* and infected with CY-17 (4 hours, scale bars = 20 μm). (**E**) Western blot confirming the knock-down of ASC or NLRP-3 with siRNAs. A further reduction in ASC expression was detected after CY-17 infection (4 hours, quantified in S8B Fig, one experiment out of 2 is shown.). (**F**) PCR amplification of a 259 bp fragment in the *MMP7* promoter (P1, -18/-276 relative to the transcription start site). (**G**) EMSA of the amplified fragment and nuclear extract from CY-17 infected HTB-9 cells (4 hours). Binding of ASC and NLRP-3 to P1 was identified as a band shift (arrow indicating protein-DNA complex). The band shift was inhibited by ASC- or NLRP-3-specific antibody. Free DNA formed a single low molecular weight band (arrow indicating free probe). The band shift was not affected by the IgG isotype control. One of three similar experiments is shown. (**H**) EMSA of the 259 bp *MMP7* promoter fragment P1 and recombinant ASC. Dose-dependent formation of an ASC-P1 complex is shown as a band shift (arrow indicating ASC-DNA complex), which was inhibited by 0.5 and 1.0 μg of anti-ASC antibodies. The band shift was not affected by negative control murine IgG control. One of three similar experiments is shown.

To understand the mechanism of increased MMP-7 expression in infected *Asc* ^-/-^ and *Nlrp3* ^*-/-*^ mice, we examined if ASC and/or NLRP-3 may act as negative regulators of *MMP7* expression. After infection of human bladder epithelial cells, with CY-17 and CY-92, we detected a significant increase in MMP-7 staining (confocal microscopy, Fig 5A and 5B). In contrast, ASC staining was reduced after infection with the virulent strains (*P* < 0.001) and NLRP-3 showed a weaker staining (*P* < 0.01). The MMP-7 response to infection and the decrease in ASC and NLRP-3 levels were confirmed by Western blot analysis (Fig 5C).

ASC or NLRP-3 expression was subsequently inhibited by transfection of human bladder epithelial cells with *ASC*- or *NLRP3-* specific siRNAs and the effects on MMP-7 expression were examined by confocal imaging (Fig 5D and S8A Fig). MMP-7 expression increased drastically in transfected and infected cells, where the expression of *ASC* or *NLRP3* had been inhibited, but not in cells transfected with negative control siRNA (Fig 5D). Inhibition efficiency of ASC and NLRP-3 expression by specific siRNAs was confirmed by Western blot analysis. Infection of the cells with CY-17 caused a further decrease in ASC and NLRP-3 staining (Fig 5E, quantified in S8B Fig). Two protein bands where detected, one of 24 kDa, corresponding to the common ASC variant and one of 20 kDa (ASC-b), corresponding to an ASC variant that enhances IL-1β secretion in human promyelocytic leukemia cells (HL60) [42] (Fig 5E).

To address if infection with cystitis strains modifies the interaction of ASC and NLRP-3 in cells, co-immunoprecipitation was performed. ASC was shown to pull down NLRP-3 in nuclear extracts of uninfected cells but after infection, a reduction in ASC/NLRP-3 interaction was detected suggesting that a loss of ASC/NLRP-3 interaction in the nuclear compartment accompanies *MMP7* activation (S8C Fig).

To determine if ASC and NLRP-3 interact with the *MMP7* promoter, DNA fragments spanning the entire promoter were used as probes in electrophoretic mobility shift assays (EMSA) (S9A Fig). A DNA fragment of 259 bp, adjacent to the transcription start site (P1, position -18/-276) was shown to interact with a nuclear protein extract from infected bladder cells, resulting in a significant band shift (Fig 5F and 5G). Specificity for ASC and NLRP-3 was confirmed by competition with specific antibodies (Fig 5G). In the absence of nuclear extract, the probe formed a single low molecular weight band, serving as a negative control. To confirm that ASC binds directly to the *MMP7* promoter, recombinant ASC protein was incubated with the 259 bp DNA sequence and examined by EMSA. Strong dose-dependent binding of ASC to *MMP7* promoter DNA was detected as a band shift, which was competitively inhibited by specific antibodies but not by the IgG isotype control (Fig 5H). Other *MMP7* promoter sequences did not interact with ASC or NLRP-3 in this assay (S9A and S9B Fig).

The results suggesting that NLRP-3 and ASC act as negative regulators of *MMP7* expression and identify an ASC binding site in *MMP7* promoter DNA, adjacent to the transcription start site.

### Therapeutic attenuation of the IL-1β response

**Fig 6:**
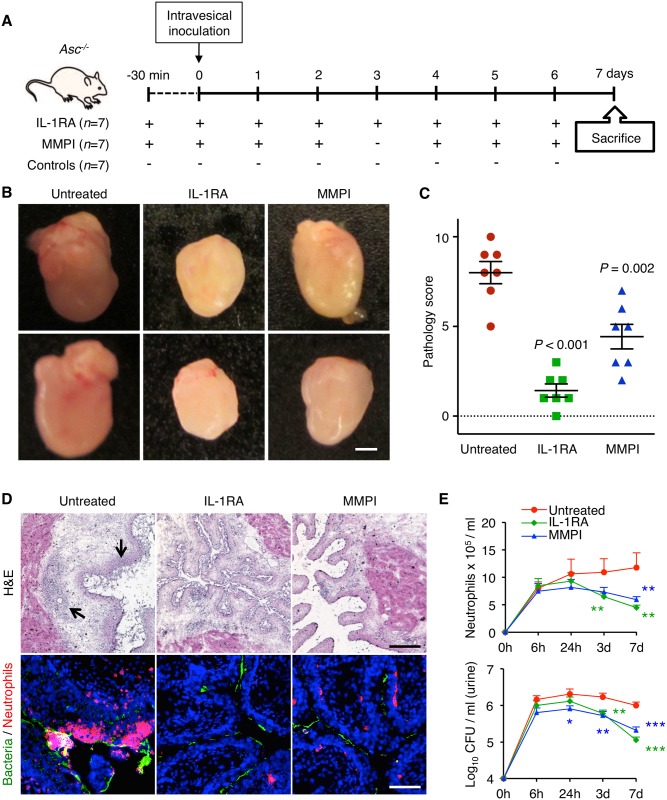
Acute cystitis immunotherapy, using an IL-1 receptor antagonist (IL-1RA) or an MMP inhibitor. (**A**) Overview of therapeutic regimen used to inhibit bladder pathology. *Asc* ^-/-^ mice were pre-treated with Anakinra (IL-1RA), 30 min before infection and daily after infection with *E*. *coli* CFT073 (1 mg in 100 μl of PBS i.p. per mouse) and sacrificed 7 days after infection. Alternatively, *Asc* ^-/-^ mice were pre-treated with the matrix metalloproteinase inhibitor (MMPI) Batimastat, 30 min before infection and daily after infection with *E*. *coli* CFT073 (0.5 mg in 100 μl of PBS i.p. per mouse, except day 3) (*n* = 7 per treatment group, total of two experiments). (**B**) Difference in gross bladder pathology between untreated controls and IL-1RA or MMPI treated mice. Two representative mice per group are shown. The IL-1RA therapy reduced macroscopic bladder pathology in *Asc* ^*-/-*^ mice. Scale bar = 1 mm. The MMPI therapy showed a similar but less pronounced effect. (**C**) Edema, hyperemia and size of the bladders were used as scoring parameters for the pathology score. Pathology scores from individual mice are shown. The gross pathology score was reduced by the inhibitors (*P* < 0.001, for IL-1RA compared to untreated *Asc* ^-/-^ mice and *P* = 0.002, for MMPI, compared to untreated *Asc* ^-/-^ mice, means ± SEMs of two experiments, two-tailed Mann Whitney test). (**D**) Protection from bladder tissue pathology shown in H&E stained sections from treated versus control mice. Arrows indicate mucosal sloughing, edema and subepithelial abscesses in untreated mice. Inhibition of mucosal neutrophil aggregate formation in bladder sections from treated mice compared to untreated and infected mice. Scale bar = 200 μm (H&E) and 50 μm (immunofluorescence). (**E**) Kinetics of neutrophil recruitment and bacterial clearance in the urine of IL-1RA or MMPI treated *Asc* ^-/-^ mice, compared to untreated mice. (*n* = 7 mice per group, means ± SEMs, * *P* < 0.05, ** *P* < 0.01, *** *P* < 0.001, two-tailed unpaired *t*-test).

To address if IL-1β serves as target for immunomodulatory therapy, we selected the most susceptible genotype (*Asc* ^*-/-*^ mice) for treatment with the IL-1 receptor antagonist (IL-1RA) Anakinra. A dose of 1 mg per mouse in 100 μl of PBS was given intra-peritoneally, 30 minutes before infection and daily after infection with *E*. *coli* CFT073 (Fig 6A). This dose was selected based on previous studies in murine models [43].

A dramatic therapeutic effect was observed compared to infected *Asc* ^*-/-*^ control mice. By macroscopic evaluation, the extent of edema, hyperemia and enlargement was reduced, resulting in a significantly lower pathology score (*P* < 0.001), (Fig 6B and 6C). By histology, a reduced inflammatory response was seen in the bladders of treated mice and mucosal pathology was inhibited compared to untreated controls that developed extensive bladder pathology (Fig 6D). Mucosal neutrophil infiltration, which accompanies pathology, was prevented and urine neutrophil numbers were low (Fig 6E). As a control for unspecific effects of Anakinra on the bacteria, CFT073 was grown in Luria-Bertani with or without 500 ng/ml of IL-1RA for 10 hours. No difference in bacterial growth rate was detected (S10 Fig).

We subsequently treated susceptible *Asc* ^*-/-*^ mice with the matrix metalloproteinase inhibitor (MMPI) Batimastat. The MMPI was given 30 minutes before infection and on days 0–2 and 4–6 after infection (0.5 mg in 100 μl of PBS i.p., Fig 6A). The MMPI had a significant protective effect (Fig 6B–6D), detected by macroscopic evaluation, resulting in a reduced pathology score (*P* = 0.002). By histology neutrophil infiltration was reduced (Fig 6D). As in the IL-1RA-treated mice, bacterial numbers remained elevated (Fig 6E). Batimastat (250 ng/ml) did not affect bacterial growth *in vitro* for up to 10 hours. No difference in bacterial growth rate was detected (S10 Fig). As Batimastat is a broad metalloproteinase inhibitor, unspecific effects on other proteases might occur. Proteases inhibited by Batimastat other than MMP-7, were not transcriptionally regulated in any of the mice with acute cystitis or controls. MMP-15, which is not susceptible to Batimastat, was weakly activated in mice with bladder pathology (FC 2.0). These findings suggest that the therapeutic effect of Batimastat reflects inhibition of MMP-7.

The results confirm the importance of IL-1β and MMP-7 for the pathogenesis of acute cystitis and identify these molecules as functional targets for immunomodulatory therapy. Bacterial counts remained elevated in the IL-1RA and the MMPI treated mice in the absence of inflammation, suggesting that the treated *Asc* ^*-/-*^ mice might develop a condition more like asymptomatic bacteriuria than acute cystitis (Fig 6E).

### IL-1β and MMP-7 responses in patients with acute cystitis

**Fig 7:**
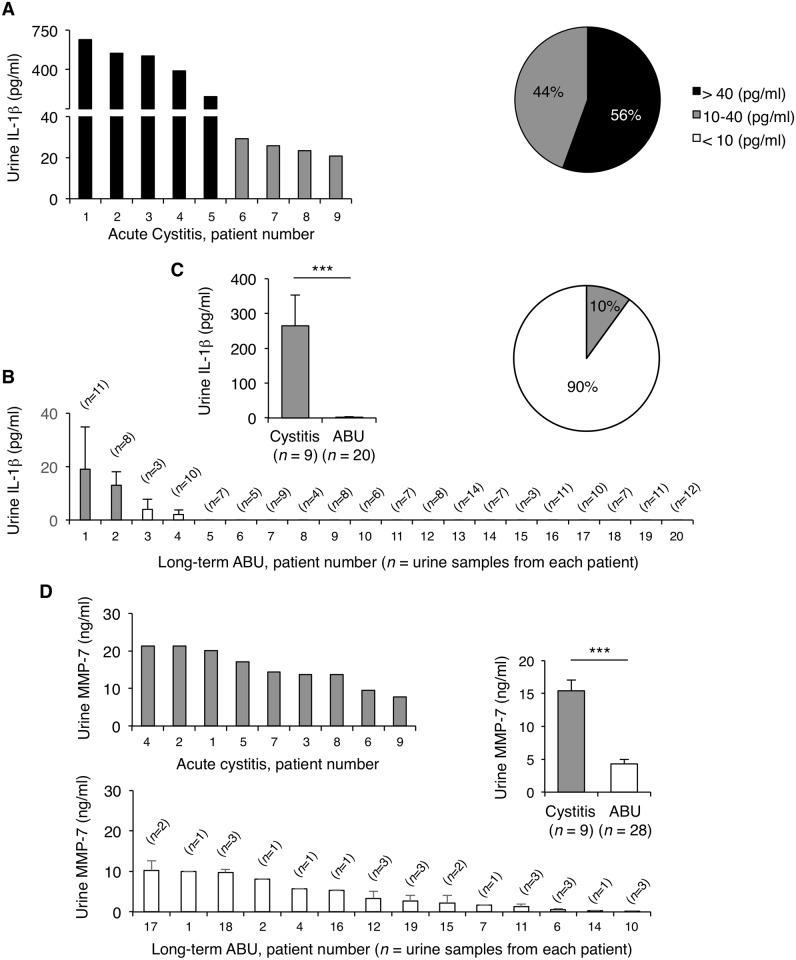
Elevated concentrations of IL-1β and MMP-7 in the urine of patients with acute cystitis. (**A**) IL-1β concentrations in urine samples from patients with acute cystitis (*n* = 9). (**B**) IL-1β concentrations in consecutive urine samples from patients with ABU, who were long-term asymptomatic carriers of *E*. *coli* 83972 [21] (means ± SEMs, 20 patients, 161 urine samples). Elevated levels of IL-1β in the cystitis patients compared to the ABU group. Pie chart (inset) depicts the distribution of IL-1β concentrations in each patient group. (**C**) Histogram compares IL-1β concentrations between the two patient groups (means ± SEMs, *** *P <* 0.001, two-tailed Mann Whitney test). (**D**) MMP-7 concentrations were higher in urine samples from the patients with acute cystitis than in patients with long-term ABU (means ± SEMs, *** *P* < 0.001, two-tailed unpaired *t*-test). Urine samples were obtained from patients with sporadic acute cystitis at the time of diagnosis. The patients with ABU participated in a prospective study of therapeutic inoculation with *E*. *coli* 83972 and were subjected to long-term follow up [21]. Multiple samples were obtained during asymptomatic carriage (3–14 samples per patient).

To examine the human relevance of the findings in the murine UTI model, we collected urine samples from patients with acute cystitis or ABU and quantified the IL-1β and MMP-7 levels, by ELISA (Fig 7). Samples from patients with sporadic episodes of acute cystitis were collected at the time of diagnosis, defined by a positive dipstick, dysuria, urgency and frequency of urination but no fever (*n* = 9). Samples were also obtained from patients with ABU (*n* = 161), who carried the prototype ABU strain *E*. *coli* 83972, following therapeutic inoculation [21]. The patients with ABU participated in a prospective study of *E*. *coli* 83972-inoculation with detailed monthly collection of symptom scores and urine samples. There were 20 patients with low symptom scores and 161 urine samples were obtained from this group.

We found elevated concentrations of IL-1β in patients with acute cystitis compared to the asymptomatic patient group (Fig 7A–7C) resulting in a means of 264.5 pg/ml and 1.5 pg/ml, respectively (*P* < 0.001).

In addition, all the patients with acute cystitis had positive MMP-7 levels, above the detection limit of 0.15 ng/ml (Fig 7D). In a subset of 28 ABU urine samples, the mean MMP-7 concentration was low, resulting in mean concentrations of 15.4 ng/ml and 4.3 ng/ml, respectively (*P* < 0.001).

The results show that patients with acute cystitis have more elevated concentrations of IL-1β and MMP-7 in urine, than patients with ABU, identifying IL-1β and MMP-7 as potential biomarkers of acute cystitis.

## Discussion

**Fig 8:**
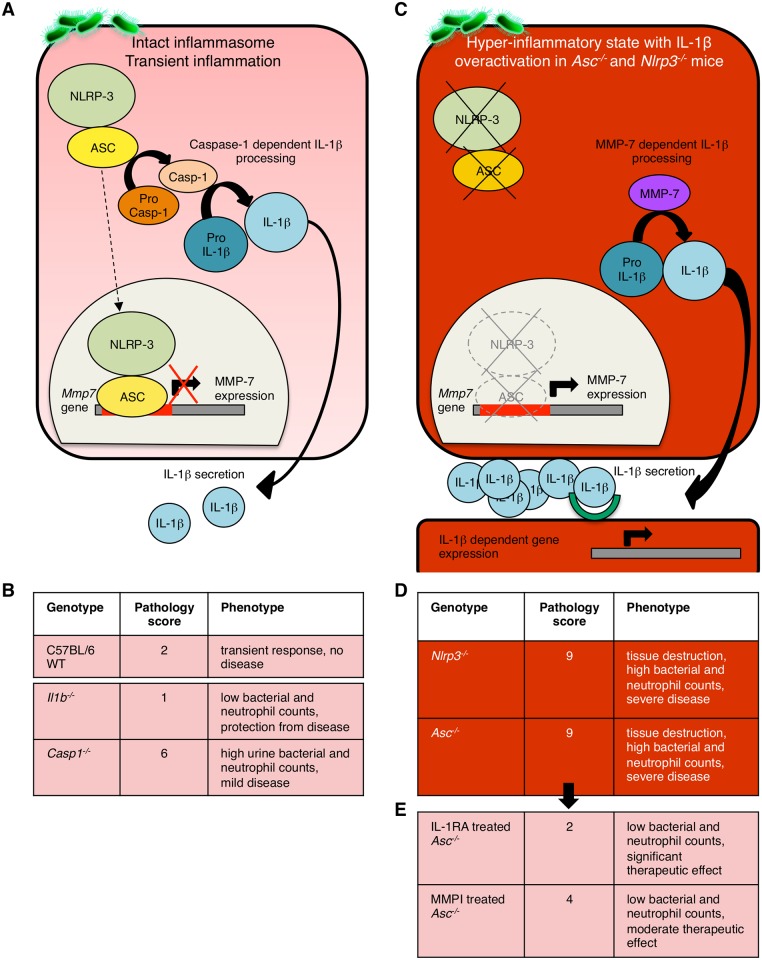
Models of IL-1β processing—cellular determinants and biological effects. (**A**) Caspase-1 dependent pro-IL-1β processing by the NLRP-3 inflammasome in mice with intact inflammasome function. The NLRP-3/ASC complex activates Caspase-1, which in turn cleaves pro-IL-1β and supports the secretion of mature IL-1β by infected cells. The production of MMP-7 is normally low, due to transcriptional repression by ASC and NLRP-3, bound to the MMP-7 promoter. (**B**) C57BL/6 WT mice develop a mild form of acute cystitis with transient inflammation. *Il1b* ^-/-^ mice were protected against infection and inflammation and *Casp1* ^*-/-*^ mice, showed an atypical phenotype without tissue damaging inflammation or neutrophil recruitment into the urine. (**C**) A new mechanism of IL-1β processing by MMP-7 in infected *Asc* ^-/-^ and *Nlrp3* ^-/-^ mice. MMP-7 over-activation in *Asc* ^-/-^ mice and *Nlrp3* ^-/-^ mice is triggered by infection and de-repression of *Mmp7* transcription. (**D**) In the absence of functional *Asc* or *Nlrp3* genes, infected mice therefore produce excessive amounts of IL-1β, causing massive bladder inflammation, with elevated neutrophil counts, edema and hypertrophy of the bladder epithelium. (**E**) Immunotherapy by suppression of the IL-1-dependent inflammatory response in susceptible *Asc* ^-/-^ mice. Mice were treated with the IL-1RA (Anakinra) or the protease inhibitor MMPI (Batimastat), which showed therapeutic effects.

Symptoms and disease are the price we pay for an efficient host defense against infection. As innate immune effectors are activated to clear tissues of bacteria, they may also cause inflammation, symptoms and tissue damage, especially if innate immune control is compromised. This is exemplified here by acute cystitis, which is a common, mostly self-limiting infection except in a subset of patients, who develop severe, recurrent infections, suggesting increased susceptibility. This study proposes a new, genetic basis of susceptibility, exemplified by the disease phenotype in *Asc* ^*-/-*^ or *Nlrp3* ^*-/-*^ mice or resistance in *Il1b* ^*-/-*^ mice that were protected from infection. The transition of the bladder mucosa from a homeostatic innate immune response to acute disease reflects the molecular control of IL-1β processing, through inflammasome-dependent or non-canonical mechanisms (Fig 8), [44–46]. The findings suggest that acute cystitis might resemble hyper-inflammatory disorders [28, 47, 48], where therapeutic efficacy of IL-1β inhibitors has been documented [49, 50]. The results provide a molecular context for acute cystitis and for the susceptibility to acute cystitis in patients with severe and chronic disease.

The severity of acute cystitis was clearly influenced by bacterial virulence as the acute cystitis strains activated IL-1β more efficiently than ABU strains. This comparison was especially valid, as the CY and ABU strains were isolated from the same pediatric population and geographic area, from children who either developed symptoms or were screened for asymptomatic carriage, by collection and culture of urine samples [31, 33]. The mechanism of IL-1β activation by the acute cystitis strains remains unclear, however. Schaale *et al*. have studied the IL-1β response of macrophages infected with UPEC strains CFT073 or UTI89 and reported that IL-1β activation and secretion is hemolysin-dependent in murine macrophages but not in human [20]. Consistent with their studies, we saw high IL-1β responses to virulent and hemolysin positive CY strains, but we did not detect a direct association with hemolysin production, suggesting that additional features control the induction and secretion of IL-1β. Schaale *at al*. also pointed to the diversity among different UPEC strains, showing that some are able to boost the inflammasome while others may escape detection by not activating IL-1β. Nagamatsu *et al*. examined hemolysin and the IL-1β response to UTI89, by inactivating a two-component signal transduction system. The mutant induced significantly higher IL-1β responses than the WT strain, in a hemolysin-dependent manner [19]. In addition, pyroptosis was linked to the presence of hemolysin, through activation of Caspase-1 and Caspase-4. In the present study, bacterial determinants of pathology were not identified but the CY isolates are being subjected to whole-genome sequence analysis for this purpose.

Paradoxically, *Il1b* ^*-/-*^ mice were resistant to infection, unlike the invasive enteropathogens *Salmonella* and *Shigella*, which are lethal for *Il1b* ^*-/-*^ mice [51, 52]. Internalization of uro-pathogens by cells in the bladder mucosa has been extensively studied and intracellular communities have been highlighted as a niche for bacterial persistence [53–55]. Specific signaling pathways involved in bacterial uptake by bladder epithelial cells include the ubiquitin-proteasome machinery [56], and especially type 1 fimbriae have been identified as essential ligands [57]. As *Il1b* ^*-/-*^ mice did not develop infection or mucosal inflammation, and proinflammatory genes were not expressed, the IL-1β response may help render the bladder mucosa susceptible to infection, possibly by enhancing bacterial growth [58] or tissue invasion. The ability to activate IL-1β production in host cells may therefore be a key to bacterial virulence, as suggested by the epidemiologic survey of strains used in the present study. The findings add MMP-7 to the list of metalloproteinases (MMP-2, MMP-3 and MMP-9) that cleave pro-IL-1β or degrade IL-1β in other cell types [59]. MMP-7 has also been shown to process and modulate the activity of anti-bacterial peptides produced by the Paneth cells in the mouse small intestine [60], where cryptdins played a protective role during *Salmonella typhimurium-* [61] or *Chlamydia trachomatis* infections [62]. In that model, pro-inflammatory effects of MMP-7 were also detected in the intestinal mucosa [63].

ASC and NLRP-3 have recently been identified as transcriptional regulators of innate immune responses. ASC forms a complex with NF-κB and modifies NF-κB-dependent gene expression [64]. NLRP-3 is involved in the T_H_2 cell differentiation program and facilitates the binding of IRF-4 to DNA [65]. Here, we identify ASC and NLRP-3 as negative regulators of *MMP7* transcription, based on 1) inverse regulation of MMP-7 with ASC and NLRP-3 in cells infected with CY-17; 2) strongly upregulated *MMP7* expression after transfection of human cells with specific siRNAs against *ASC* and *NLRP3*; 3) identification of a specific *MMP7* promoter DNA sequence, to which nuclear proteins from infected cells bind; 4) inhibition by antibodies to ASC or NLRP-3 of the interaction between nuclear proteins and the *MMP7* promoter; 5) binding of recombinant ASC to the *MMP7* promoter. As HDAC6 was recently found to interact with NLRP-3 and to modulate its inflammasome function [66], we speculate that NLRP-3 may act as a co-repressor by binding to ASC and recruiting histone deacetylase (HDAC) to the *MMP7* promoter, thereby generating a tight chromatin structure refractory to transcription. This was supported by evidence of a direct interaction between ASC and NLRP-3 in the nuclei of CY-17 infected cells. Future studies are required to address in greater detail the regulation of *MMP7* expression by ASC and NLRP-3.

Acute cystitis is a handicap, socially and emotionally but despite its prevalence and importance for patients and society, acute cystitis is a poorly understood disease [6, 67]. Social and behavioral factors have been emphasized as a cause of recurrent infections and until recently, therapeutic options have included a variety of shorter or longer antibiotic regimens, many of which have been discontinued, due to resistance development. It comes as no surprise, that this highly painful condition has been the focus of various interventions in addition to antibiotic therapy. Deliberate establishment of competitive microflora has shown promising clinical effects [21, 22] but novel, therapeutic approaches are needed in this large patient group. In this study, we show that acute cystitis is amenable to IL-1 receptor inhibition and/or MMP blockade. As IL-1RA is in clinical use, short-term immunotherapy might be a realistic option as an adjunct to antibiotics in acute cystitis patients. The identified molecular disease determinants may also be helpful to address the unmet need for diagnostic tools in this patient group. The frequency of genetic variants, such as *ASC* mutations, and their relevance to disease would be an interesting focus of prospective clinical studies.

## Materials and Methods

### Bacterial strains and cell infection procedure

Cystitis (CY) and asymptomatic bacteriuria (ABU) strains were prospectively isolated during a study of childhood UTI in Göteborg, Sweden, using standard microbiological techniques [32, 33]. The strain collection has been extensively studied and characterized with fimbrial genotype and phenotype, virulence factor expression, OKH antigen profiles and multilocus enzyme typing [31, 68]. The hemolytic activity was assessed with blood agar plates, where the hemolytic zone surrounding the central stab of bacteria is recorded. The phenotype has been compared to the *hly* genotype and found to be a very close fit. The UPEC strain, *E*. *coli* CFT073 (O6:K2:H1) [69] and the ABU strain *E*. *coli* 83972 (OR:K5:H-) [25] have been extensively characterized, including whole genome sequencing and were used as positive or negative controls. Bacteria were cultured on tryptic soy agar (TSA, 16 h, 37°C), harvested in phosphate-buffered saline (PBS, pH 7.2) and diluted as appropriate in RPMI without FCS. In the screen of IL-1β responses to acute cystitis or ABU strains, cells were exposed to 10^8^ CFU/ml of bacteria with Gentamicin for 4 hours. In remaining experiments, cells were exposed to 10^5^ CFU/ml for 1 hour or 4 hours without antibiotics. Overnight static cultures of *E*. *coli* CFT073, CY-17 or CY-92 or 83972 in Luria-Bertani (LB) broth were used for experimental infection.

### Cell culture

Human bladder grade II carcinoma cells (5637, ATCC# HTB-9) were cultured, to 70–80% confluency on 8-well chamber Permanox slides (6x10^4^ cells/well), in 6-well plates (6x10^5^ cells/well) or 96-well plates (5x10^4^ cells/well), (all from Thermo Scientific) in RPMI-1640 supplemented with 1 mM sodium pyruvate, 1 mM non-essential amino acids and 10% heat-inactivated FBS (PAA) at 37°C with 5% CO_2_. Gentamicin (50 μg/ml) was from GE Healthcare.

### Cell viability assay

HTB-9 cells in 96-well plates were infected for 1h or 4h. PrestoBlue (Invitrogen, A13262) was added to each well to a final concentration of 10%. After 20 min of incubation at 37°C, total well fluorescence was measured using a microplate reader Infinite F200 (Tecan), with 585 nm excitation and 620 nm emission filters.

### Cytokine measurements

IL-1β concentrations in filtered supernatants (Syringe Filter w/0.2 μm PES, VWR) from cells infected with 10^8^ CFU/ml (with Gentamicin, 4 hours) were determined by Immulite 1000 (Siemens) and IL-1β concentrations in cell supernatants or urine by Human or Mouse IL-1β/IL-1F2 DuoSet ELISA kits (all from R&D Systems). Urine MMP-7 levels were quantified with Human total MMP-7 Immunoassay Quantikine ELISA (R&D Systems).

### Confocal microscopy

Cells were infected, fixed (3.7% formaldehyde, 10 min), permeabilized (0.25% Triton X-100, 5% FBS, 15 min), blocked (5% FBS, 1h at RT), incubated with primary antibodies in 5% FBS overnight at 4°C (anti-IL-1 beta, 1:100, ab9722; anti-MMP7, 1:25, ab4044, all Abcam; anti-ASC, 1:50, sc-22514-R, Santa Cruz; anti-NLRP3/Cryo-2, 1:100, AG-20B-0014-C100, Adipogen) and appropriate secondary antibody (Alexa Fluor 488 goat anti-rabbit IgG, A-11034, or goat anti-mouse IgG, A-11001; Life Technologies), (1h at RT). After nuclear staining (DRAQ5, Abcam), slides were mounted (Fluoromount, Sigma-Aldrich), imaged by laser-scanning confocal microscopy (LSM510 META confocal microscope, Carl Zeiss) and quantified by ImageJ software 1.46r (NIH).

### Western blotting

Cells were lysed with RIPA lysis buffer, supplemented with protease and phosphatase inhibitors (both from Roche Diagnostics) and fractionated using the NE-PER Nuclear and Cytoplasmic extraction reagents (Thermo Scientific). Supernatants were filtered and concentrated by trichloroacetic acid precipitation, followed by aceton desiccation. Proteins were run on SDS-PAGE (4–12% Bis-Tris gels, Invitrogen), blotted onto PVDF membranes (GE Healthcare) blocked with 5% bovine serum albumin (BSA) or non-fat dry milk (NFDM), incubated with primary antibody: rabbit anti-IL-1 beta (1:2,500 in 5% NFDM, ab9722, Abcam), rabbit anti-ASC (1:200 in 5% BSA, sc-22514-R, Santa Cruz), mouse anti-NLRP3/NALP3 (1:1,000 in 5% milk, Cryo-2, Adipogen) or rabbit anti-MMP7 (1:200 in 5% BSA, ab4044, Abcam), washed with PBS tween 0.1% and incubated with secondary antibodies in 5% NFDM (goat anti rabbit-HRP or goat anti-mouse-HRP, Cell Signaling). Bands were imaged using ECL Plus detection reagent (GE Health Care) and quantified using ImageJ. GAPDH (1:1,000, sc-25778, Santa Cruz) was used as loading control.

### Co-immunoprecipitation

Nuclear fractions, extracted as described in **Western blotting**, were incubated with rabbit anti-ASC antibody (sc-22514-R, Santa Cruz, 1 μg/ml) overnight and complexes were collected with magnetic Dynabeads Protein G (Life technologies), analyzed by SDS-PAGE with rabbit anti-ASC and mouse anti-NLRP3 (Cryo-2, Adipogen) primary antibodies (1:200–1:1,000, 5% BSA), followed by secondary anti-rabbit (Cell Signaling) or anti-mouse (DAKO) antibodies (1:4,000, 5% NFDM).

### Global gene expression

Total RNA was extracted from murine bladders or kidneys in RLT buffer with 1% β-Mercaptoethanol after disruption in a tissue homogenizer (TissueLyser LT, Qiagen) using Precellys Lysing kits (Bertin Technologies), with the RNeasy Mini Kit (Qiagen), 100 ng of RNA was amplified using GeneChip 3´IVT Express Kit, 6 μg of fragmented and labeled aRNA was hybridized onto Mouse Genome 430 PM array strips for 16 hours at 45°C, washed, stained and scanned using the Geneatlas system (all Affymetrix). All samples passed the internal quality controls included in the array strips (signal intensity by signal to noise ratio; hybridization and labeling controls; sample quality by GAPDH signal and 3’-5’ ratio < 3).

Transcriptomic data was normalized using Robust Multi Average implemented in the Partek Express Software (Partek) [70, 71]. Fold change was calculated by comparing infected (7 days) to uninfected mice of the same genetic background. Significantly altered genes were sorted by relative expression (2-way ANOVA model using Method of Moments, *P*-values < 0.05 and absolute fold change > 1.41) [38]. Heat-maps were constructed by Gitools 2.1.1 software. Differentially expressed genes and regulated pathways were analyzed by Ingenuity Pathway Analysis software (IPA, Ingenuity Systems, Qiagen). Qiagen’s list of 84 key inflammasome genes was selected for analysis.

The microarray data are available in the NCBI’s Gene Expression Omnibus repository (accession number GSE86096).

### *In vitro* proteolysis

Recombinant human IL-1β, NLRP-3 or PYCARD (ASC) (280 ng, H00003553-P02, H00114548-P01 or H00029108-P01, Abnova) were incubated with recombinant active human MMP-7 (0.035U, #444270 Merck Millipore) in MMP reaction buffer (20 mM Tris, pH 7.6, 5 mM CaCl_2_, 0.1 M NaCl) at 37°C until stopped with 100 mM DDT. Fragments were detected by Western blot, using rabbit anti-IL-1 beta (1:2 000, ab9722, Abcam), rabbit anti-ASC (1:1 000, p9522-75, US Biological) and rabbit anti-NLRP3 (1:500, sc-66846, Santa Cruz).

### IL-1β activity assay

HTB-9 cells were treated with the products of the *in vitro* proteolysis of pro-IL-1β by MMP-7 at different concentrations or with pro-IL-1β or MMP-7 alone, serving as negative controls. Prostaglandin E2 (PGE_2_) concentrations were measured in filtered supernatants (Syringe Filter w/0.2 μm PES, VWR) by ELISA (R&D systems).

### siRNA transfection

HTB-9 cells were transfected with *PYCARD*/*ASC* and *NLRP3* specific siRNAs (0.09 μM, FlexiTube GeneSolution, #GS29108 and #GS114548, Qiagen) or with AllStars Negative Control siRNA (#SI03650318, Qiagen) using the HiPerFect Transfection Reagent (#301705, Qiagen) for 17 hours, then infected. Transfection efficiency was assessed by Western blotting.

### PCR analysis

*MMP7* promoter and promoter flanks were amplified in 10 different fragments by PCR using 15 ng of total human genomic DNA. For forward and reverse primers (http://primer3.ut.ee/), see S3 Table. Thermal cycling conditions were as follows: 95°C for 2 min, 35 cycles (95°C for 30 s, 60°C for 30 s and 72°C for 40 s) and 72°C for 5 min.

### Electrophoretic mobility shift assay (EMSA)

Amplified DNA sequences from the *MMP7* promoter were used as probes and labeled with GelGreen (Biotium). Each reaction contained 3–5 μg of DNA probe with, 5 μg of nuclear extract from infected HTB-9 cells, or 0.2–0.65 μg recombinant ASC (Abnova, H00029108-P01) or NLRP-3 (Abnova, H00114548-P01) in binding buffer (100 mM Tris, 500 mM NaCl and 10 mM DTT, pH 7). For the band shift competition assay, 0.5–1 μg of rabbit anti-ASC (Santa Cruz, sc-22514-R,) or 0.5 μg of rabbit anti-NLRP3 (Cryo-2, Adipogen) antibodies were used. Binding reactions were incubated at 15°C for 30 min, loaded onto a 6% non-denaturing, non-reducing polyacrylamide gel and ran in a 50 mM Tris (pH 7), 0.38 M glycine, and 2 mM EDTA buffer at 100 V for 2–3 hours. Mouse IgG2A isotype control (R&D Systems, MAB003) was used as negative control antibody. Gels were imaged using the Bio-RAD ChemiDoc system.

### Experimental urinary tract infection

Mice were bred and housed in the specific pathogen-free MIG animal facilities (Lund, Sweden) with free access to food and water. Female C57BL/6 mice or *Il1b* ^-/-^ [36], *Nlrp3* ^-/-^ [34] *Asc* ^-/-^ [35], *Casp1* ^-/-^ [37], *Mmp7* ^*-/-*^ [40] mice were used at 9–15 weeks of age. The *Il1b* ^-/-^ mice have recently been shown to be functionally defective for IL-1α [72]. The *Casp1* ^-/-^ mice were also deficient for Caspase-11 [73]. *Nlrp3* ^*-/-*^ and *Asc* ^-/-^ mice were from Jürg Tschopp's laboratory, Department of Biochemistry, University of Lausanne and Institute for Arthritis Research (aIAR). *Mmp7* ^-/-^ and *Casp1* ^-/-^ mice were purchased from The Jackson Laboratories, USA.

Mice were intravesically infected under Isofluorane anesthesia (10^8^ CFU in 0.1 ml), through a soft polyethylene catheter (outer diameter 0.61 mm; Clay Adams). Animals were sacrificed under anesthesia; bladders and kidneys were aseptically removed and macroscopic pathology was documented by photography. Tissues were fixed with 4% paraformaldehyde or frozen for sectioning and RNA extraction. Viable counts in homogenized tissues (Stomacher 80, Seward Medical) were determined on TSA (37°C, overnight). Urine samples were collected prior to and at regular times after infection and quantitatively cultured. Neutrophils in uncentrifuged urine were counted, using a hemocytometer.

Gross pathology was scored based on the macroscopic appearance of the bladders at sacrifice. The score was based on edema, hyperemia and size, on a scale of 0–10, where 0 is unchanged compared to the uninfected controls and 10 is most edematous, most hyperemic and largest size.

### Histology and immunohistochemistry

Tissues were embedded in O.C.T. compound (VWR) and 5-μm-thick fresh cryosections on positively charged microscope slides (Superfrost/Plus; Thermo Scientific) were fixed with 4% paraformaldehyde or acetone-methanol (1:1 v/v). For H&E or immunohistochemistry, sections were blocked and permeabilized (0.2% Triton X-100, 5% goat normal serum (DAKO) or 1% BSA (Sigma), stained (anti-neutrophil antibody [NIMP-R14] (ab2557, Abcam), polyclonal *E*. *coli* antibody (1:100, NB200-579, Novus Biologicals), anti-IL-1 beta (1:50, ab9722, Abcam) or anti-MMP-7 (1:100, ab4044, Abcam), all rabbit antibodies). Alexa 488 anti-rat IgG or anti-rabbit IgG and Alexa 568 anti-rabbit IgG (A-21210, A-11001 and A-11011, Life Technologies) were secondary antibodies and nuclei were counterstained with DAPI (0.05 mM, Sigma-Aldrich). Imaging was by fluorescence microscopy (AX60, Olympus Optical). Richard-Allan Scientific Signature Series Hematoxylin 7211 and Eosin-Y 7111 (Thermo Scientific) were used to counterstain the tissue sections.

Histology was scored using H&E stained bladder sections. The score was based on neutrophil infiltration, tissue architecture and epithelial thickness on a scale of 0–10, where 0 is unchanged compared to uninfected controls and 10 the highest neutrophil infiltration, most destroyed tissue architecture and maximum epithelial thickness.

### IL-1β and MMP-7 therapy

The IL-1 receptor antagonist, Anakinra (Kineret, SOBI) or the broad-spectrum MMP inhibitor, Batimastat (ab142087, Abcam) were injected intraperitoneally (i.p.) as described in Fig 6A.

### Patients

Urine samples from patients with sporadic acute cystitis were obtained at two primary care clinics in Lund, Sweden. A diagnosis of acute cystitis was based on a urine dipstick analysis positive for bacteria and symptoms from the lower urinary tract, including frequency, dysuria and suprapubic pain. Midstream urine specimens were obtained at the time of diagnosis.

Patients with ABU were included in a placebo-controlled study of asymptomatic bacteriuria, following intravesical inoculation with *E*. *coli* 83972 [21]. Briefly, *E*. *coli* 83972 bacteriuria was established by intravesical inoculation (10^5^ CFU/ml in saline), daily for three days and the outcome was measured as the total number of UTIs during an optimal period of 12 months followed by a cross over to a similar period without *E*. *coli* 83972 bacteriuria. Urine samples were obtained for cytokine analysis during *E*. *coli* 83972 bacteriuria, with negative symptom scores [21].

### Statistics

ELISA results, fluorescence intensity, Pathology, bacterial numbers and neutrophil responses were analyzed by unpaired two-tailed *t*-test or Mann-Whitney test after assessment of normality with the d’Agostino & Pearson omnibus normality test. Significance was accepted at *P* < 0.05 (*), *P* < 0.01 (**) or *P* < 0.001 (***). For animal numbers, see S1 Table. Data was examined using Prism (v. 6.02, GraphPad).

### Ethics statement

Experimental infections were approved by the Malmö/Lund Animal Experimental Ethics Committee at the Lund District Court in Sweden (approval number M44-13). All animal care and protocols were governed by the European Parlement and Council Directive 2010/63/EU, the Swedish Animal Welfare Act (Djurskyddslag 1988:534), the Swedish Animal Welfare Ordinance (Djurskyddsförordning 1988:539) and Institutional Animal Care and Use Committee (IACUC) guidelines. Experiments were reported according to the ARRIVE guidelines. The clinical studies were approved by the Human Ethics Committee at Lund University (approval numbers LU106-02, LU236-99 and Clinical Trial Registration RTP-A2003, International Committee of Medical Journal Editors, www.clinicaltrials.gov). Patients gave their informed written consent.

## Supporting Information

S1 Fig Cell viability assay. Human bladder carcinoma (HTB-9) cells were infected with CY-17, CY-92, CY-132 and CY-49, CFT073 or ABU for 1 hour or 4 hours. Cell viability was measured by PrestoBlue assay. Cell viability was > 95% after 1 hour and > 90% after 4 hours.
(TIF)

S2 Fig Uninfected bladders and IL-1β secretion. (**A**) Bladder morphology of uninfected mice from *Asc* ^-/-^, *Nlrp3* ^-/-^ and *Il1b* ^-/-^ genotypes. Scale bars = 1 mm. (**B**) Urine IL-1β concentrations, followed from 6 hours to 7 days after infection with CFT073, quantified by ELISA (left panel), *n* = 6–7 mice per group, means ± SEMs, * *P* < 0.05, ** *P* < 0.01, *** *P* < 0.001, unpaired Mann Whitney test, compared to C57BL/6 WT mice. Western blot of IL-1β in urine samples obtained after 7 days (right panel).
(TIF)

S3 Fig Bladder pathology in *Asc* ^-/-^ mice infected with acute cystitis strains CY-17 or CY-92. No pathology in *Asc* ^-/-^ mice infected the ABU strain *E*. *coli* 83972. (**A**) Dramatic increase in size, compared to uninfected controls, with general hyperemia and protruding edematous areas. (**B**) Inflammation and tissue destruction (H&E stained sections). Scale bar = 100 μm. (**C**) Strong bacterial- and neutrophil staining detected by immunohistochemistry. Scale bar = 50 μm. (**D**) Elevated bacterial and neutrophil counts in urine (*n* = 4 mice per group, means ± SEMs). (**E**) No evidence of disease in *Asc* ^-/-^ mice infected with the ABU strain *E*. *coli* 83972 or in C57BL/6 WT mice, shown by gross bladder pathology and neutrophil counts in urine after 24 hours and 7 days (*n* = 5 mice per group). See also S1 Table.
(TIF)

S4 Fig *Casp1* ^-/-^ mice are protected from tissue damage but show a macroscopic response to infection. *Casp1* ^-/-^ mice were infected with CFT073 and sacrificed after 7 days. *Asc* ^-/-^ mice were used as positive controls for bladder pathology. In contrast to *Asc* ^-/-^ mice, *Casp1* ^-/-^ mice did not develop bladder tissue pathology. (**A**) Intact tissue structure by H&E staining and no evidence of inflammatory cell infiltration. Few neutrophils and bacteria were detected in the tissues, by immunohistochemistry. Scale bars = 50 μm. (**B**) Bladder edema and hyperemia in *Casp1* ^-/-^ mice infected with CFT073. Less pronounced response than in *Asc* ^-/-^ mice (see Fig 2A). Scale bar = 1 mm. (**C**) Elevated bacterial- and neutrophil counts in urine of *Casp1* ^-/-^ mice and *Asc* ^*-/-*^ mice (Exp 3 in S1 Table, 5 mice per group). Neutrophils were elevated in urine of both mouse strains. (**D**) Elevated bacterial counts in tissue samples. The elevated bacterial numbers in *Casp1* ^-/-^ and *Asc* ^-/-^ mice suggested that a functional inflammasome is essential for bacterial clearance from infected bladders. (**E**) Detection of IL-1b by immunohistochemistry of bladder tissue sections. Massive retention of IL-1b in the bladder mucosa of *Casp1* ^-/-^ mice but not in *Asc* ^-/-^ mice. Scale bar = 50 μm. (**F**) Secretion of IL-1b into the urine in *Asc* ^-/-^ and *Casp1* ^-/-^ mice, detected by ELISA. Urine IL-1b levels were low in *Casp1* ^-/-^ mice (*n* = 5, means ± SEMs, ** *P* < 0.01, *** *P* < 0.001 compared to *Asc* ^-/-^ mice, unpaired t-test). (**G**) Lack of inflammasome gene activation in infected *Casp1* ^-/-^ mice compared to *Asc* ^-/-^ mouse (7d).
(TIF)

S5 Fig Transcriptomic regulation during infection. (**A**) Top regulated pathways in mice with bladder pathology. Gene expression analysis comparing whole bladder RNA from *Asc* ^-/-^ and *Nlrp3* ^-/-^ mice with severe acute cystitis to protected *Il1b* ^-/-^ mice and C57BL/6 WT mice with mild bladder inflammation (Ingenuity Pathway Analysis). Bars show the -log(*P*-value) of the submitted gene list. (**B**) Lack of IL-1β dependent gene expression in the kidneys of infected C57BL/6 WT mice.
(TIF)

S6 Fig MMP-7 staining in mice bladder tissue. (**A**) Lack of MMP-7 staining in uninfected control mice. (**B**) Separate MMP-7 and neutrophil staining in infected bladder tissue (24 h). Immunohistochemistry of bladder sections obtained 24 hours after infection of *Asc* ^-/-^ mice with CFT073. MMP-7 (red) was detected in the epithelium and recruited neutrophils (green) were present throughout with increased density towards the lumen. In most areas with recruited neutrophils, MMP-7 co-localization was not detected. Scale bar = 50 μm.
(TIF)

S7 Fig Processing by MMP-7 of IL-1β, ASC and NLRP-3. (**A**) Western blot analysis of the time-dependent cleavage of GST-tagged pro-IL-1β by MMP-7, using an antibody to mature IL-1β. Arrows indicate mature IL-1b (18 kDa) and a 16 kDa band. Weak staining of the GST-tagged pro-IL-1β. (**B**) Time-dependent degradation of ASC by MMP-7. (**C**) NLRP-3 was not cleaved by MMP-7 and was used as a negative control.
(TIF)

S8 Fig Controls for Fig 5. (**A**) MMP-7 immunofluorescence staining of uninfected HTB-9 cells transfected with *NLRP3* and *ASC* siRNA. In the absence of infection, *NLRP3* siRNA or *ASC* siRNA did not increase MMP-7 expression. (**B**) Quantification of the Western blot in Fig 5E. Each band was normalized against its corresponding GAPDH band. (**C**) Co-immunoprecipitation of nuclear extracts with anti-ASC antibodies. Pull-down of NLRP-3 is detected in control cells but attenuated in CY-17 infected cells.
(TIF)

S9 Fig Mapping of the amplified P1 fragment in the *MMP7* gene promoter. (**A**) The human *MMP7* gene is located on chromosome 11 q22.3. Ensemble GRCh37 (release 81, July 2015) sequence of chromosome 11 region 102,391,471–102,421,479 including the *MMP7* gene and promoter. Transcription factor binding sites were identified using the Champion ChiP Transcription Factor Search Portal. Various primers were designed to map the promoter and the two promoter flanking regions. DNA sequences were amplified by PCR and used for EMSA (Fig 5F–5H). (**B**) EMSA using different DNA fragments from the *MMP7* promoter and ASC or NLRP-3 recombinant proteins. Binding was only seen with the P1 fragment and ASC protein, arrow (see Fig 5H).
(TIF)

S10 Fig IL-1RA or MMPI did not influence bacterial growth. Bacterial growth in Luria-Bertani (LB) broth in the presence of Anakinra (500 ng/ml) or Batimastat (250 ng/ml) for 10 hours. No significant effect was observed.
(TIF)

S1 Table Number of mice used for experimental infection, specified for each group of experiments. The total number of mice was 147.
(PDF)

S2 Table Genes regulated in mice with pathology compared to mice without pathology. (PDF)

S3 Table Primers used to amplify the *MMP7* promoter and promoter flanks. (PDF)
