# Supplementary figures and images for: Molecular Basis of Acute Cystitis Reveals Susceptibility Genes and Immunotherapeutic Targets
Source: PLoS Pathog. 2016 Oct 12;12(10):e1005848. doi: 10.1371/journal.ppat.1005848 (PMC5061333; doi:10.1371/journal.ppat.1005848)

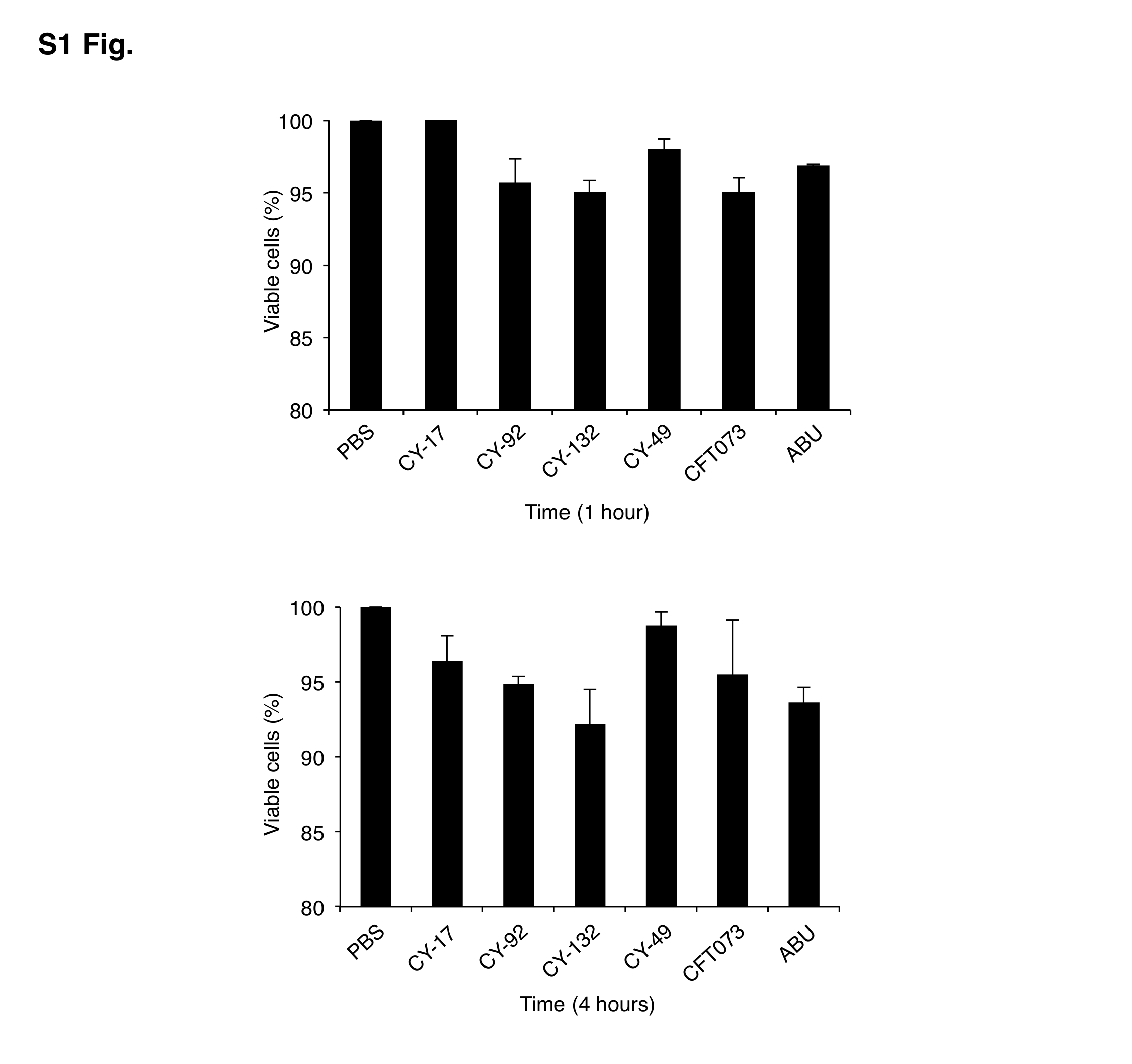

Supplement: S1 Fig — Human bladder carcinoma (HTB-9) cells were infected with CY-17, CY-92, CY-132 and CY-49, CFT073 or ABU for 1 hour or 4 hours. Cell viability was measured by PrestoBlue assay. Cell viability was > 95% after 1 hour and > 90% after 4 hours. (TIF) [file ppat.1005848.s001.tif]

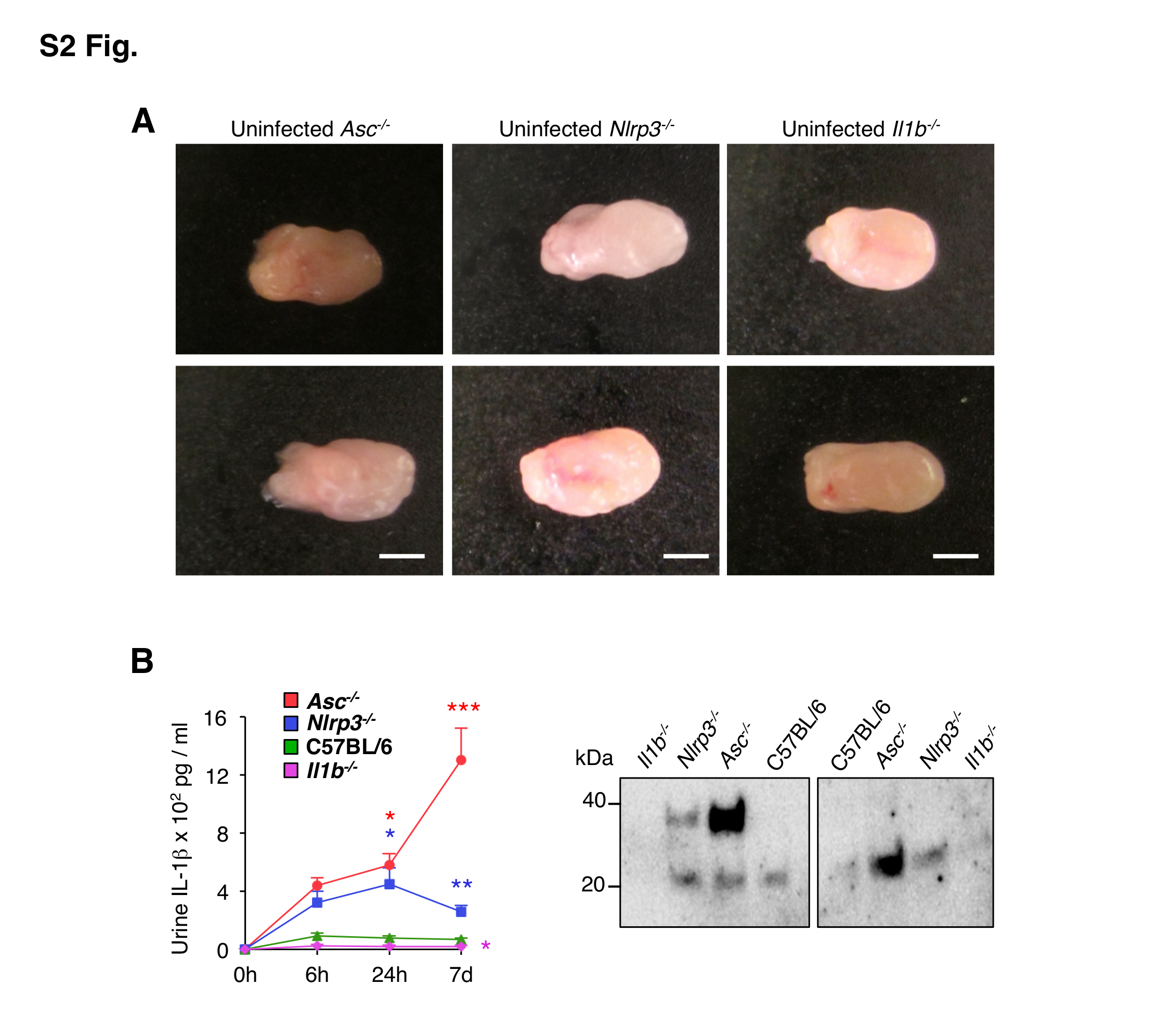

Supplement: S2 Fig — (A) Bladder morphology of uninfected mice from Asc -/-, Nlrp3 -/- and Il1b -/- genotypes. Scale bars = 1 mm. (B) Urine IL-1β concentrations, followed from 6 hours to 7 days after infection with CFT073, quantified by ELISA (left panel), n = 6–7 mice per group, means ± SEMs, * P < 0.05, ** P < 0.01, *** P < 0.001, unpaired Mann Whitney test, compared to C57BL/6 WT mice. Western blot of IL-1β in urine samples obtained after 7 days (right panel). (TIF) [file ppat.1005848.s002.tif]

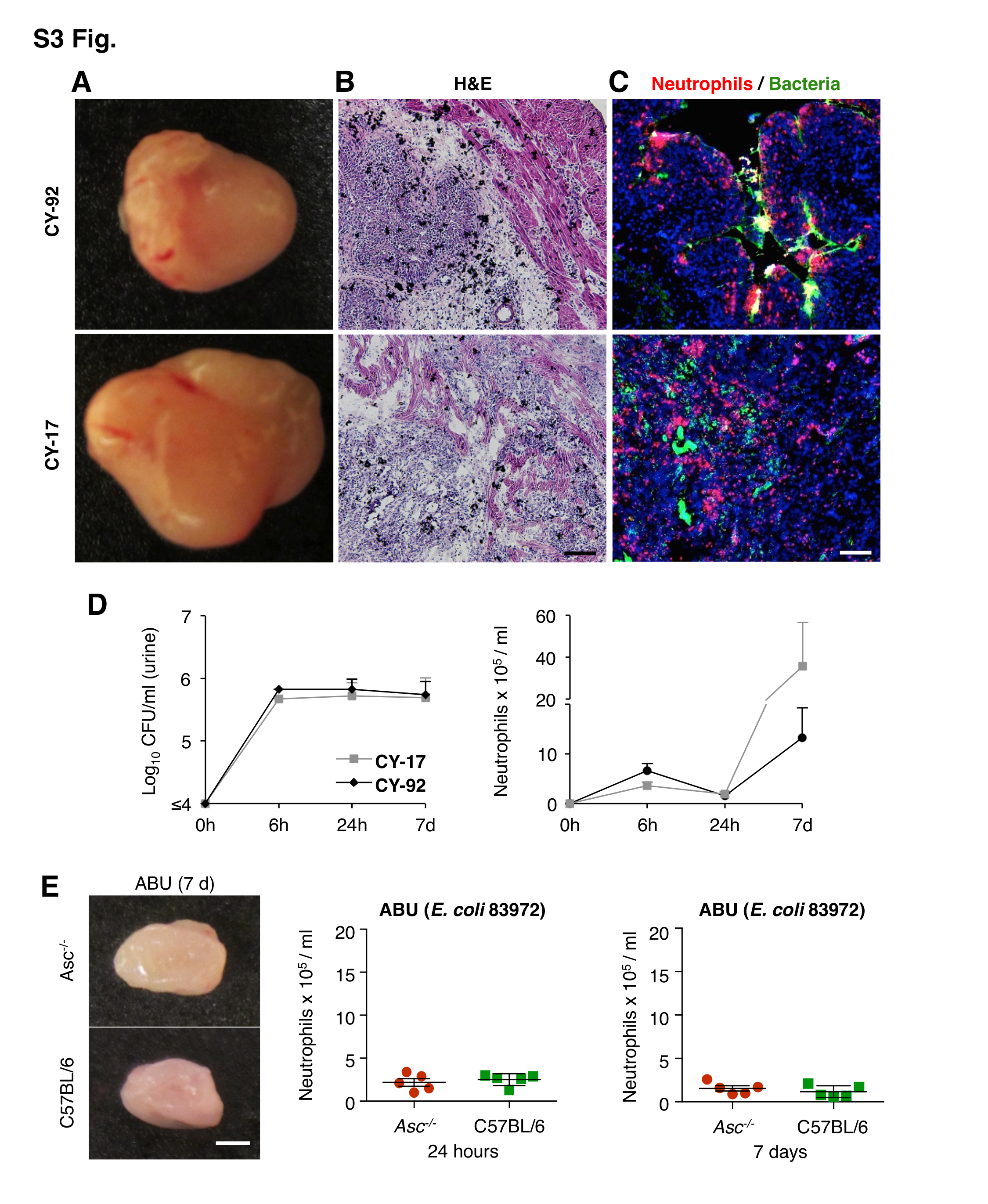

Supplement: S3 Fig — No pathology in Asc -/- mice infected the ABU strain E. coli 83972. (A) Dramatic increase in size, compared to uninfected controls, with general hyperemia and protruding edematous areas. (B) Inflammation and tissue destruction (H&E stained sections). Scale bar = 100 μm. (C) Strong bacterial- and neutrophil staining detected by immunohistochemistry. Scale bar = 50 μm. (D) Elevated bacterial and neutrophil counts in urine (n = 4 mice per group, means ± SEMs). (E) No evidence of disease in Asc -/- mice infected with the ABU strain E. coli 83972 or in C57BL/6 WT mice, shown by gross bladder pathology and neutrophil counts in urine after 24 hours and 7 days (n = 5 mice per group). See also S1 Table. (TIF) [file ppat.1005848.s003.tif]

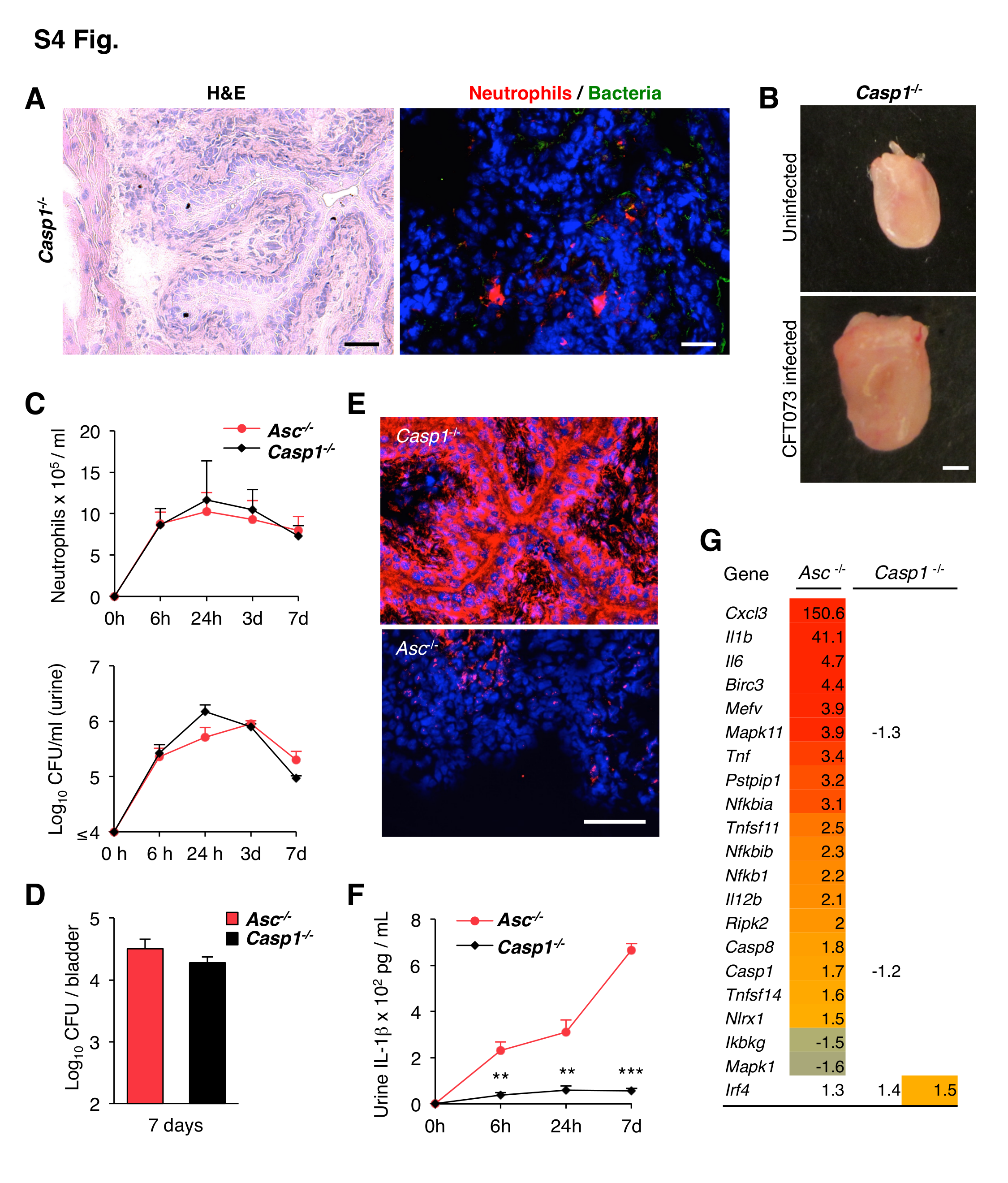

Supplement: S4 Fig — Casp1 -/- mice were infected with CFT073 and sacrificed after 7 days. Asc -/- mice were used as positive controls for bladder pathology. In contrast to Asc -/- mice, Casp1 -/- mice did not develop bladder tissue pathology. (A) Intact tissue structure by H&E staining and no evidence of inflammatory cell infiltration. Few neutrophils and bacteria were detected in the tissues, by immunohistochemistry. Scale bars = 50 μm. (B) Bladder edema and hyperemia in Casp1 -/- mice infected with CFT073. Less pronounced response than in Asc -/- mice (see Fig 2A). Scale bar = 1 mm. (C) Elevated bacterial- and neutrophil counts in urine of Casp1 -/- mice and Asc -/- mice (Exp 3 in S1 Table, 5 mice per group). Neutrophils were elevated in urine of both mouse strains. (D) Elevated bacterial counts in tissue samples. The elevated bacterial numbers in Casp1 -/- and Asc -/- mice suggested that a functional inflammasome is essential for bacterial clearance from infected bladders. (E) Detection of IL-1b by immunohistochemistry of bladder tissue sections. Massive retention of IL-1b in the bladder mucosa of Casp1 -/- mice but not in Asc -/- mice. Scale bar = 50 μm. (F) Secretion of IL-1b into the urine in Asc -/- and Casp1 -/- mice, detected by ELISA. Urine IL-1b levels were low in Casp1 -/- mice (n = 5, means ± SEMs, ** P < 0.01, *** P < 0.001 compared to Asc -/- mice, unpaired t-test). (G) Lack of inflammasome gene activation in infected Casp1 -/- mice compared to Asc -/- mouse (7d). (TIF) [file ppat.1005848.s004.tif]

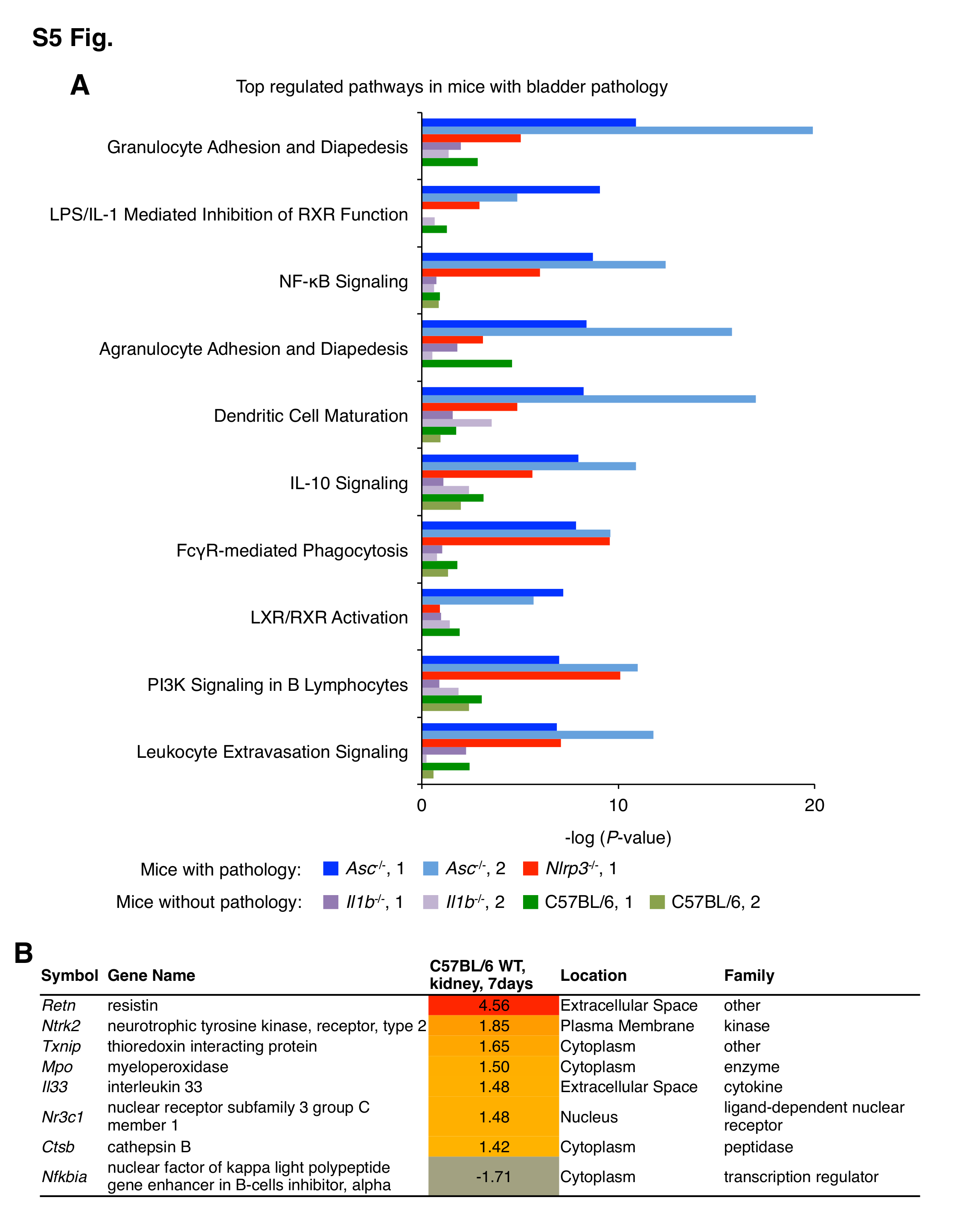

Supplement: S5 Fig — (A) Top regulated pathways in mice with bladder pathology. Gene expression analysis comparing whole bladder RNA from Asc -/- and Nlrp3 -/- mice with severe acute cystitis to protected Il1b -/- mice and C57BL/6 WT mice with mild bladder inflammation (Ingenuity Pathway Analysis). Bars show the -log(P-value) of the submitted gene list. (B) Lack of IL-1β dependent gene expression in the kidneys of infected C57BL/6 WT mice. (TIF) [file ppat.1005848.s005.tif]

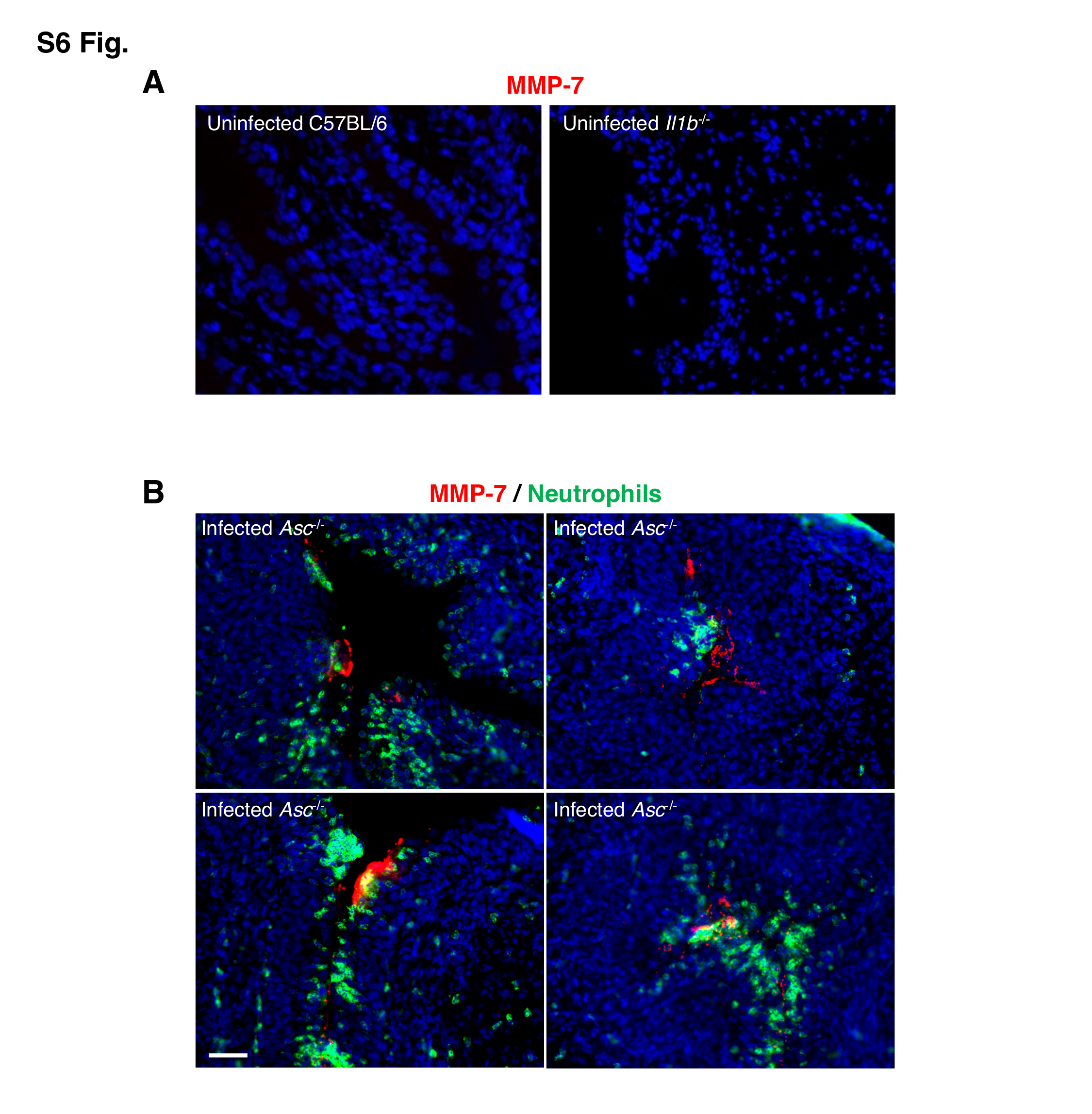

Supplement: S6 Fig — (A) Lack of MMP-7 staining in uninfected control mice. (B) Separate MMP-7 and neutrophil staining in infected bladder tissue (24 h). Immunohistochemistry of bladder sections obtained 24 hours after infection of Asc -/- mice with CFT073. MMP-7 (red) was detected in the epithelium and recruited neutrophils (green) were present throughout with increased density towards the lumen. In most areas with recruited neutrophils, MMP-7 co-localization was not detected. Scale bar = 50 μm. (TIF) [file ppat.1005848.s006.tif]

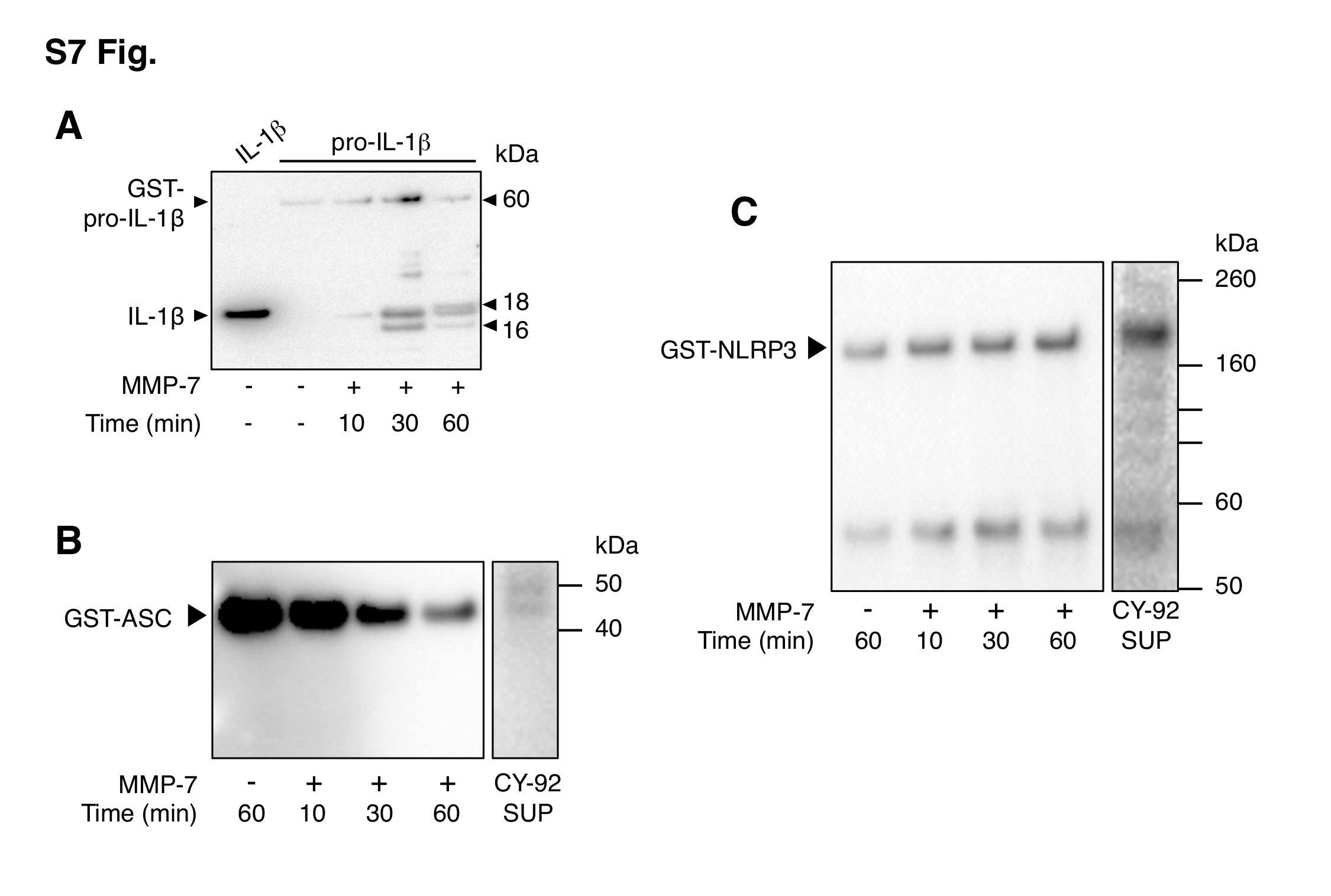

Supplement: S7 Fig — (A) Western blot analysis of the time-dependent cleavage of GST-tagged pro-IL-1β by MMP-7, using an antibody to mature IL-1β. Arrows indicate mature IL-1b (18 kDa) and a 16 kDa band. Weak staining of the GST-tagged pro-IL-1β. (B) Time-dependent degradation of ASC by MMP-7. (C) NLRP-3 was not cleaved by MMP-7 and was used as a negative control. (TIF) [file ppat.1005848.s007.tif]

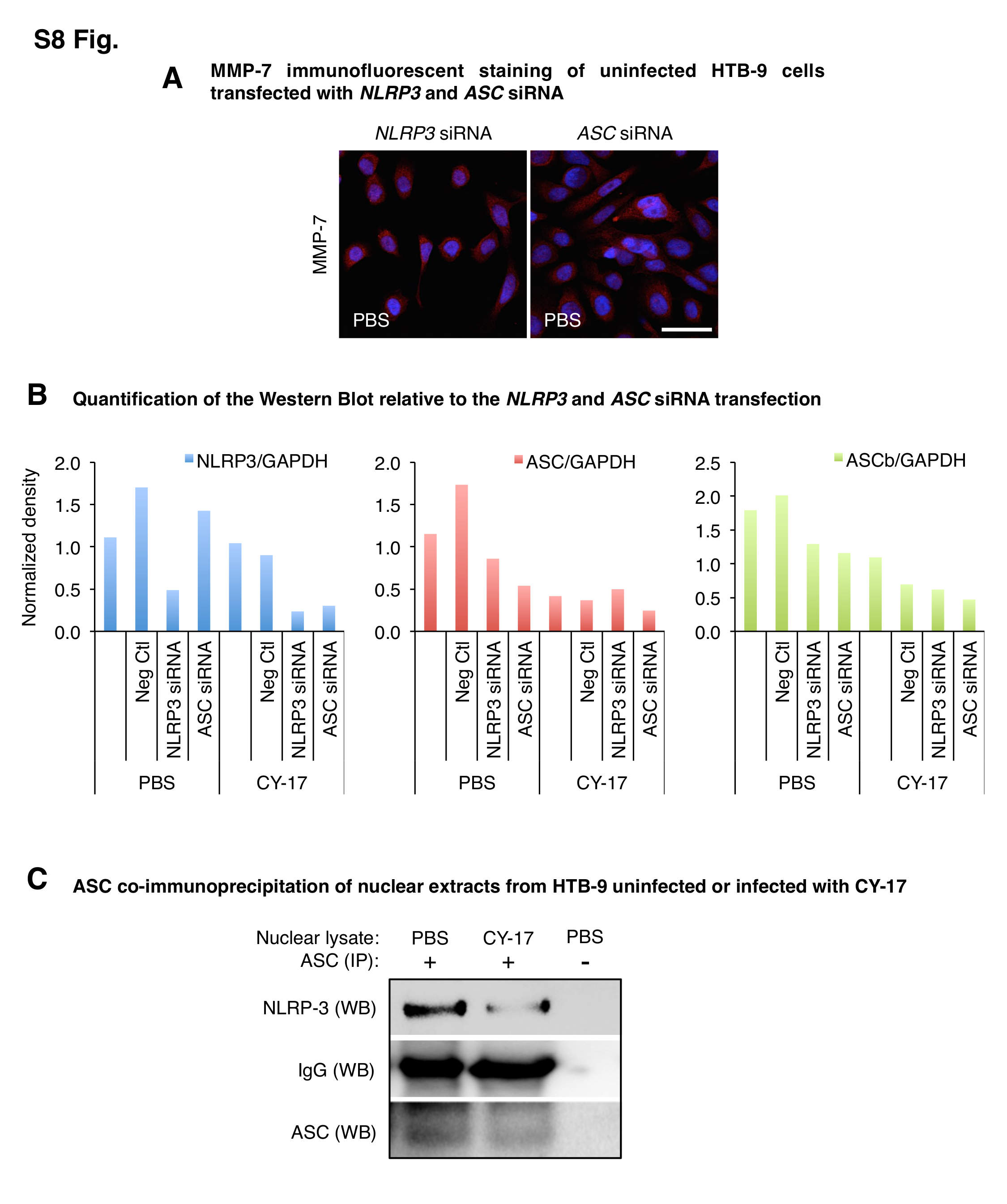

Supplement: S8 Fig — (A) MMP-7 immunofluorescence staining of uninfected HTB-9 cells transfected with NLRP3 and ASC siRNA. In the absence of infection, NLRP3 siRNA or ASC siRNA did not increase MMP-7 expression. (B) Quantification of the Western blot in Fig 5E. Each band was normalized against its corresponding GAPDH band. (C) Co-immunoprecipitation of nuclear extracts with anti-ASC antibodies. Pull-down of NLRP-3 is detected in control cells but attenuated in CY-17 infected cells. (TIF) [file ppat.1005848.s008.tif]

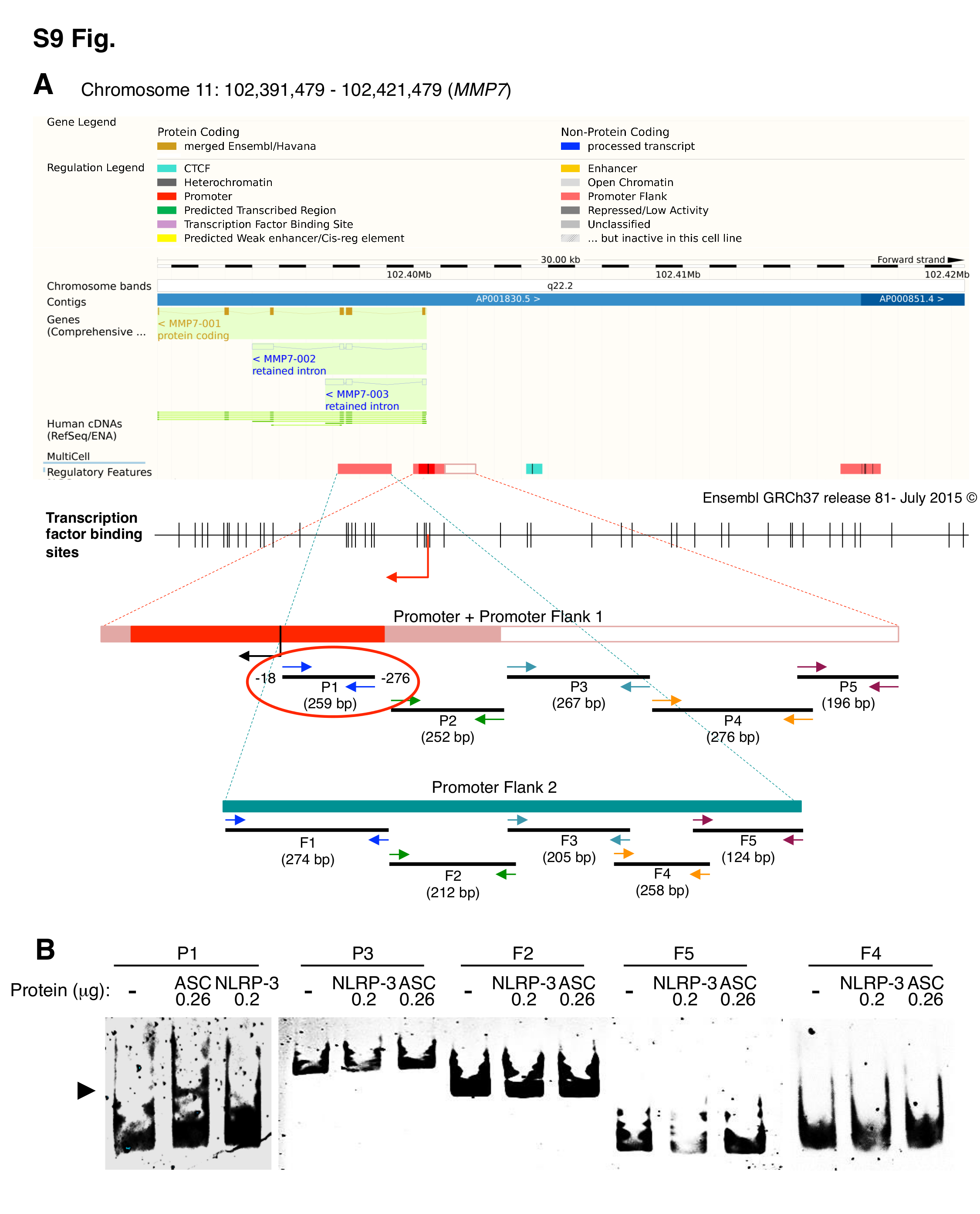

Supplement: S9 Fig — (A) The human MMP7 gene is located on chromosome 11 q22.3. Ensemble GRCh37 (release 81, July 2015) sequence of chromosome 11 region 102,391,471–102,421,479 including the MMP7 gene and promoter. Transcription factor binding sites were identified using the Champion ChiP Transcription Factor Search Portal. Various primers were designed to map the promoter and the two promoter flanking regions. DNA sequences were amplified by PCR and used for EMSA (Fig 5F–5H). (B) EMSA using different DNA fragments from the MMP7 promoter and ASC or NLRP-3 recombinant proteins. Binding was only seen with the P1 fragment and ASC protein, arrow (see Fig 5H). (TIF) [file ppat.1005848.s009.tif]

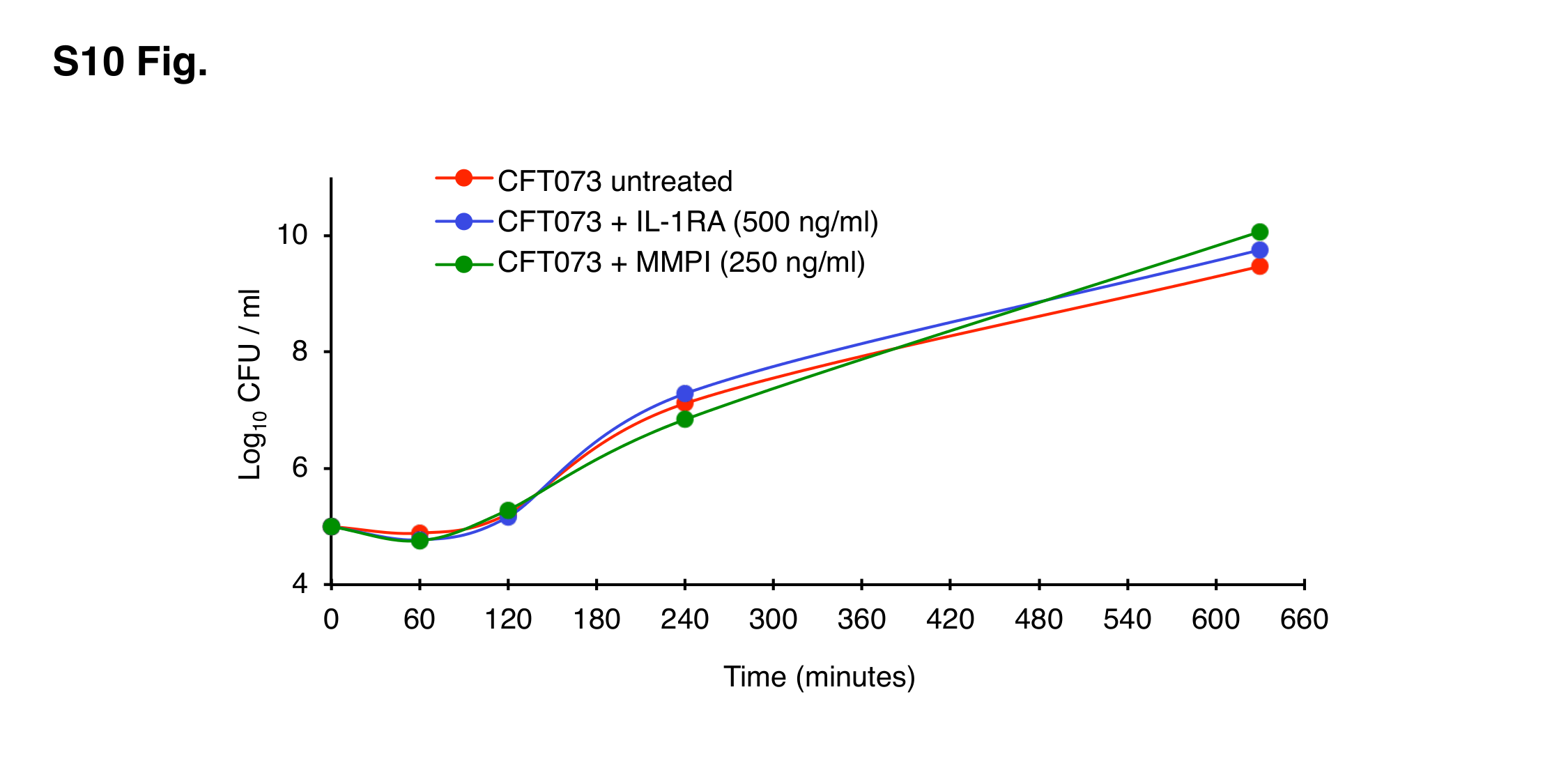

Supplement: S10 Fig — Bacterial growth in Luria-Bertani (LB) broth in the presence of Anakinra (500 ng/ml) or Batimastat (250 ng/ml) for 10 hours. No significant effect was observed. (TIF) [file ppat.1005848.s010.tif]
