# Supplementary material for: Molecular Basis of Acute Cystitis Reveals Susceptibility Genes and Immunotherapeutic Targets
Source: PLoS Pathog. 2016 Oct 12;12(10):e1005848. doi: 10.1371/journal.ppat.1005848 (PMC5061333; doi:10.1371/journal.ppat.1005848)
Supplement: S1 Table — The total number of mice was 147. (PDF) [file ppat.1005848.s011.pdf]

S1 Table. Number of mice used for experimental infection, specified for each group of experiments. The total number of mice was 147.

Data in Fig 2. Genotype screen; CFT073 infection

**Experiment 1**	***Asc**$^{-/-}$*	***Nlrp3**$^{-/-}$*	**C57BL/6**	
Uninfected	2	2	2	Samples collected & sacrificed
6 h	4	4	3	Samples collected & sacrificed
24 h	4	4	3	Samples collected & sacrificed
3 d	4	5	5	Urine collected
7 d	4	5	5	Samples collected & sacrificed

**Experiment 2**	***Asc**$^{-/-}$*	***Nlrp3**$^{-/-}$*	**C57BL/6**	***Il1b**$^{-/-}$*	
Uninfected	3	3	4	3	Samples collected & sacrificed
6 h	10	6	6	10	Urine collected
24 h	10	6	6	10	Urine collected
3 d	10	6	6	10	Urine collected
7 d	10	6	6	10	Samples collected & sacrificed

Data in S3 Fig. Acute cystitis strains; *Asc*$^{-/-}$ mice; CY-17 and CY-92 infection

	**CY-17**	**CY-92**	
6 h	4	4	Urine collected
24 h	4	4	Urine collected
7 d	4	4	Samples collected & sacrificed

Data in S3 Fig. C57BL/6 and *Asc*$^{-/-}$ mice; ABU infection

	**C57BL/6**	***Asc**$^{-/-}$*	
24 h	5	5	Urine collected
7 d	5	5	Samples collected & sacrificed

Data in S4 Fig. Genotype screen; *Mmp7*$^{-/-}$, *Casp1*$^{-/-}$ and *Asc*$^{-/-}$ mice; CFT073 infection

**Experiment 3**	***Mmp7**$^{-/-}$*	***Casp1**$^{-/-}$*	***Asc**$^{-/-}$*	
Uninfected	2	2	2	Samples collected & sacrificed
6 h	5	5	5	Urine collected
24 h	5	5	5	Urine collected
3 d	5	5	5	Urine collected
7 d	5	5	5	Samples collected & sacrificed

Data in Fig 6. Therapy; *Asc*$^{-/-}$ mice; CFT073 infection

	Untreated	IL-1RA	MMPI	
6 h	4	4	3	Urine collected
24 h	4	4	3	Urine collected
3 d	4	4	3	Urine collected
7 d	4	4	3	Samples collected & sacrificed

	Untreated	IL-1RA	MMPI	
6 h	3	3	4	Urine collected
24 h	3	3	4	Urine collected
3 d	3	3	4	Urine collected
7 d	3	3	4	Samples collected & sacrificed
