# Supplementary material for: Molecular Basis of Acute Cystitis Reveals Susceptibility Genes and Immunotherapeutic Targets
Source: PLoS Pathog. 2016 Oct 12;12(10):e1005848. doi: 10.1371/journal.ppat.1005848 (PMC5061333; doi:10.1371/journal.ppat.1005848)
Supplement: S2 Table — (PDF) [file ppat.1005848.s012.pdf]

S2 Table. Genes regulated in mice with pathology compared to mice without pathology (blue = genes with FC < -1.41; red = genes with FC > 1.41)

Symbol	Entrez Gene Name	*Asc*$^{-/-}$ 1	*Asc*$^{-/-}$ 2	*Nlrp3*$^{-/-}$ 1	*Il1b*$^{-/-}$ 1	C57BL/6
Mmp7	matrix metallopeptidase 7 (matrilysin, uterine)	239.87	124.02	259.28	-1.16	1.08
Ubd	ubiquitin D	150.06	204.89	210.56	1.04	-1.03
Cxcl6	chemokine (C-X-C motif) ligand 6	95.41	240.12	232.12	1.20	1.03
Cxcl1	chemokine (C-X-C motif) ligand 1 (melanoma growth stimulating activity, alpha)	72.61	197.62	136.80	1.19	1.11
S100a8	S100 calcium binding protein A8	56.89	89.87	124.45	1.17	1.09
Cxcl3	chemokine (C-X-C motif) ligand 3	55.87	162.92	98.95	2.14	1.13
Olfm4	olfactomedin 4	33.53	10.88	32.14	-1.05	1.13
Lcn2	lipocalin 2	28.46	109.61	40.64	1.70	1.11
S100a9	S100 calcium binding protein A9	25.99	36.33	46.88	1.08	-1.14
Saa3	serum amyloid A 3	14.77	17.76	16.54	-1.16	-2.06
Adam8	ADAM metallopeptidase domain 8	14.61	18.43	22.30	1.25	1.18
Pigr	polymeric immunoglobulin receptor	13.86	13.53	27.80	1.79	1.23
Arg1	arginase 1	13.75	1.85	32.89	-1.02	1.01
Clec6a	C-type lectin domain family 6, member A	13.48	14.56	7.49	1.01	-1.07
Ltb	lymphotoxin beta (TNF superfamily, member 3)	10.60	22.11	12.19	3.02	1.68
Il1b	interleukin 1, beta	9.54	38.59	11.67	1.07	1.14
Spp1	secreted phosphoprotein 1	7.69	2.53	7.18	-1.18	-1.04
Fpr2	formyl peptide receptor 2	7.47	12.62	12.28	1.00	1.13
Aif1	allograft inflammatory factor 1	6.40	9.86	7.53	1.57	1.20
Cd14	CD14 molecule	6.02	10.43	9.49	1.44	1.46
Slpi	secretory leukocyte peptidase inhibitor	5.75	4.26	2.36	-1.44	-2.57
Irg1	immunoresponsive gene 1	5.45	9.29	7.15	1.40	1.17
Slfn2	schlafen 2	5.42	7.60	9.81	1.62	1.14
Bcl3	B-cell CLL/lymphoma 3	5.36	10.33	8.57	1.61	1.34
Il33	interleukin 33	5.22	8.18	10.61	2.23	-1.08
Serpina3	serpin peptidase inhibitor, clade A (alpha-1 antiproteinase, antitrypsin), member 3	4.89	3.34	4.19	-1.50	-1.18
Ppbp	pro-platelet basic protein (chemokine (C-X-C motif) ligand 7)	4.36	1.51	5.71	1.06	-1.02
Cfb	complement factor B	4.22	6.07	3.75	1.13	-1.56
Sftpd	surfactant protein D	4.16	1.41	2.46	-1.14	-1.06
Tgtp1/ Tgtp2	T cell specific GTPase 1	4.07	7.50	4.99	1.50	-1.52
Cxcr4	chemokine (C-X-C motif) receptor 4	4.01	4.80	5.28	1.32	1.51
Selplg	selectin P ligand	4.00	4.88	3.77	1.46	1.32
Cyba	cytochrome b-245, alpha polypeptide	4.00	5.17	3.66	1.21	1.06
Ccl5	chemokine (C-C motif) ligand 5	3.99	11.43	9.76	4.12	2.25
Fcer1g	Fc fragment of IgE, high affinity I, receptor for; gamma polypeptide	3.93	5.66	5.24	1.00	-1.38
Cerk	ceramide kinase	3.93	2.94	3.32	1.28	1.10
Tlr1	toll-like receptor 1	3.83	5.31	4.96	1.49	-1.02
Relb	v-rel avian reticuloendotheliosis viral oncogene homolog B	3.80	6.41	3.75	1.39	1.27
Hk2	hexokinase 2	3.73	7.38	7.89	1.65	2.11
Tmem173	transmembrane protein 173	3.71	5.01	4.86	1.16	1.04
Ncf4	neutrophil cytosolic factor 4, 40kDa	3.58	3.53	4.15	1.35	1.09
Clec7a	C-type lectin domain family 7, member A	3.56	4.80	3.58	1.44	1.22
Elf3	E74-like factor 3 (ets domain transcription factor, epithelial-specific)	3.48	4.24	6.46	1.42	1.35
Tyrobp	TYRO protein tyrosine kinase binding protein	3.34	4.89	3.74	-1.10	-1.39
Ikbke	inhibitor of kappa light polypeptide gene enhancer in B-cells, kinase epsilon	3.31	4.74	3.20	2.12	1.77
Ifi47	interferon gamma inducible protein 47	3.27	6.15	5.65	1.21	-1.47
Icam1	intercellular adhesion molecule 1	3.24	5.50	2.34	1.34	-1.03
Srgn	serglycin	3.24	5.03	4.52	-1.05	-1.16
Ifit1b	interferon-induced protein with tetratricopeptide repeats 1B	3.20	9.85	9.91	1.34	-1.75
Tlr2	toll-like receptor 2	3.10	4.29	2.97	1.10	1.03
Apoc2	apolipoprotein C-II	3.08	2.43	4.41	-1.05	-1.05
Pstpip1	proline-serine-threonine phosphatase interacting protein 1	3.07	3.25	3.43	1.06	1.20
Fcgr2a	Fc fragment of IgG, low affinity IIa, receptor (CD32)	3.04	3.46	2.94	-1.23	-2.02
Wfdc17	WAP four-disulfide core domain 17	3.02	3.76	2.80	-1.55	-1.49
Ctss	cathepsin S	3.00	3.55	5.06	1.37	1.17
Socs3	suppressor of cytokine signaling 3	2.93	7.44	4.80	1.21	-1.08
Gch1	GTP cyclohydrolase 1	2.93	4.26	2.82	1.13	1.13
Ifit3	interferon-induced protein with tetratricopeptide repeats 3	2.92	8.50	4.91	1.01	-1.52
Irf7	interferon regulatory factor 7	2.83	3.89	2.17	1.07	-1.33
Mefv	Mediterranean fever	2.82	3.46	2.71	1.01	1.07
Arrb2	arrestin, beta 2	2.77	3.39	2.84	1.32	-1.01
Ccl9	chemokine (C-C motif) ligand 9	2.76	3.52	2.89	-1.40	-1.80

Symbol	Entrez Gene Name	*Asc*$^{-/-}$ 1	*Asc*$^{-/-}$ 2	*Nlrp3*$^{-/-}$ 1	*Il1b*$^{-/-}$ 1	C57BL/6
Il1rn	interleukin 1 receptor antagonist	2.72	3.03	1.53	-1.02	1.03
Csf2rb	colony stimulating factor 2 receptor, beta, low-affinity (granulocyte-macrophage)	2.70	4.03	2.00	1.11	1.05
Egr1	early growth response 1	2.64	4.53	1.37	2.14	-1.77
Timp1	TIMP metallopeptidase inhibitor 1	2.62	2.83	1.91	-1.23	-1.10
Cd300lf	CD300 molecule-like family member f	2.54	2.79	3.29	1.01	-1.06
Xdh	xanthine dehydrogenase	2.52	2.63	2.60	-1.19	-1.36
Casp4	caspase 4, apoptosis-related cysteine peptidase	2.49	3.57	3.26	1.55	1.36
Ctsc	cathepsin C	2.48	3.27	2.54	-1.01	1.02
Irf1	interferon regulatory factor 1	2.38	3.60	3.70	1.37	-1.30
C3	complement component 3	2.37	2.78	2.46	-1.54	-1.43
Mmp8	matrix metallopeptidase 8 (neutrophil collagenase)	2.33	1.21	1.64	-1.05	1.06
Tlr13	toll-like receptor 13	2.32	1.94	2.28	-1.07	-1.02
Hbegf	heparin-binding EGF-like growth factor	2.30	2.98	3.11	1.35	-1.04
Tnfaip8l2	tumor necrosis factor, alpha-induced protein 8-like 2	2.26	2.66	1.60	-1.06	1.00
Nfkbie	nuclear factor of kappa light polypeptide gene enhancer in B-cells inhibitor, epsilon	2.20	3.47	1.22	-1.05	1.09
Procr	protein C receptor, endothelial	2.20	3.06	1.65	1.37	1.18
Gbp2	guanylate binding protein 2	2.19	3.92	2.27	-1.04	-2.26
Ccl2	chemokine (C-C motif) ligand 2	2.17	5.92	2.30	1.48	-1.13
Ccl3l1/ Ccl3l3	chemokine (C-C motif) ligand 3-like 1	2.16	5.90	2.95	-1.00	1.00
Prdm1	PR domain containing 1, with ZNF domain	2.15	4.49	3.27	1.41	1.14
Map3k8	mitogen-activated protein kinase kinase kinase 8	2.15	2.33	1.79	1.07	1.12
Ptpn22	protein tyrosine phosphatase, non-receptor type 22 (lymphoid)	2.13	5.63	2.52	1.28	1.13
Serpinb1	serpin peptidase inhibitor, clade B (ovalbumin), member 1	2.13	2.82	3.05	1.04	-1.61
Cd24a	CD24a antigen	2.07	2.15	3.06	1.19	1.16
C3ar1	complement component 3a receptor 1	2.07	1.94	1.73	1.03	-1.05
Ier3	immediate early response 3	2.03	2.47	2.32	1.62	1.05
Acp5	acid phosphatase 5, tartrate resistant	2.01	1.86	4.05	-1.12	-1.24
Hp	haptoglobin	2.01	4.02	39.22	1.45	1.00
Psmb9	proteasome (prosome, macropain) subunit, beta type, 9	2.00	3.30	2.01	1.14	-1.16
Ccl17	chemokine (C-C motif) ligand 17	2.00	16.03	1.36	1.26	1.91
Apoe	apolipoprotein E	1.97	2.43	2.48	-1.45	-1.17
S100a3	S100 calcium binding protein A3	1.96	1.37	1.04	1.12	1.01
Tnf	tumor necrosis factor	1.96	3.83	3.88	-1.11	1.00
Il18	interleukin 18 (interferon-gamma-inducing factor)	1.94	1.79	1.18	-1.10	-1.55
Pf4	platelet factor 4	1.94	1.08	1.48	-1.71	-2.00
Kdr	kinase insert domain receptor (a type III receptor tyrosine kinase)	1.92	2.22	2.21	1.26	1.17
Ube2l6	ubiquitin-conjugating enzyme E2L 6	1.91	3.94	2.36	1.23	-1.13
Ifngr1	interferon gamma receptor 1	1.91	2.12	1.92	1.07	-1.14
Pilrb	paired immunoglobin-like type 2 receptor beta	1.89	2.61	2.35	-1.23	-1.16
Itgam	integrin, alpha M (complement component 3 receptor 3 subunit)	1.87	1.59	2.22	-1.11	-1.23
Tmem176b	transmembrane protein 176B	1.86	1.75	1.38	-1.28	-1.04
Nfkbia	nuclear factor of kappa light polypeptide gene enhancer in B-cells inhibitor, alpha	1.84	2.43	1.56	1.02	-1.05
Nfil3	nuclear factor, interleukin 3 regulated	1.84	2.66	3.57	1.32	2.15
Nfkb1	nuclear factor of kappa light polypeptide gene enhancer in B-cells 1	1.84	2.30	1.74	1.04	1.18
F7	coagulation factor VII (serum prothrombin conversion accelerator)	1.82	1.28	1.82	-1.01	-1.04
Psmb10	proteasome (prosome, macropain) subunit, beta type, 10	1.81	2.90	1.83	-1.06	-1.23
Igfbp3	insulin-like growth factor binding protein 3	1.81	1.82	2.07	1.05	-1.22
Cd274	CD274 molecule	1.80	4.74	4.00	1.11	-1.31
Cxcl10	chemokine (C-X-C motif) ligand 10	1.79	23.63	6.72	3.07	-1.43
Cd69	CD69 molecule	1.78	6.04	2.36	1.27	-1.03
Hgfac	HGF activator	1.78	1.38	1.20	1.21	1.12
Lyn	v-yes-1 Yamaguchi sarcoma viral related oncogene homolog	1.76	2.07	1.55	1.07	1.01
Traf2	TNF receptor-associated factor 2	1.76	2.23	1.53	1.07	1.09
Ccl20	chemokine (C-C motif) ligand 20	1.75	3.07	1.62	1.10	1.28
Myd88	myeloid differentiation primary response 88	1.75	2.26	1.92	1.24	1.05
Pld3	phospholipase D family, member 3	1.71	1.48	1.76	1.06	1.20
Itgax	integrin, alpha X (complement component 3 receptor 4 subunit)	1.71	1.89	1.73	1.32	-1.04
Krt19	keratin 19	1.69	1.48	1.62	1.06	1.07
Il18r1	interleukin 18 receptor 1	1.69	2.59	1.61	1.73	1.23
Cd74	CD74 molecule, major histocompatibility complex, class II invariant chain	1.68	3.48	2.03	1.29	-1.03
Lcp1	lymphocyte cytosolic protein 1 (L-plastin)	1.68	2.13	1.84	1.34	1.06

Symbol	Entrez Gene Name	*Asc*$^{-/-}$ 1	*Asc*$^{-/-}$ 2	*Nlrp3*$^{-/-}$ 1	*Il1b*$^{-/-}$ 1	C57BL/6
Il36a	interleukin 36, alpha	1.67	1.43	1.24	-1.09	-1.09
Nlrx1	NLR family member X1	1.67	1.75	2.34	1.38	1.18
Casp12	caspase 12 (gene/pseudogene)	1.64	1.71	1.14	-1.32	-1.39
S100a4	S100 calcium binding protein A4	1.63	2.22	1.16	1.13	-1.04
Ido1	indoleamine 2,3-dioxygenase 1	1.62	5.02	10.09	1.14	-1.18
Il1a	interleukin 1, alpha	1.61	5.34	2.75	-1.11	1.05
Csf2	colony stimulating factor 2 (granulocyte-macrophage)	1.61	8.20	3.05	-1.06	-1.09
Ptger4	prostaglandin E receptor 4 (subtype EP4)	1.60	2.13	1.63	1.11	1.18
Rps6ka1	ribosomal protein S6 kinase, 90kDa, polypeptide 1	1.60	1.45	1.65	1.35	1.22
Birc2	baculoviral IAP repeat containing 2	1.60	1.89	2.00	1.36	1.15
Trem3	triggering receptor expressed on myeloid cells 3	1.60	1.72	1.59	-1.06	1.25
Tap2	transporter 2, ATP-binding cassette, sub-family B (MDR/TAP)	1.59	2.01	2.21	1.13	1.12
Cd83	CD83 molecule	1.59	5.14	1.54	1.55	1.13
Rsad2	radical S-adenosyl methionine domain containing 2	1.59	2.40	1.09	1.01	1.11
Psme2	proteasome (prosome, macropain) activator subunit 2 (PA28 beta)	1.58	2.55	1.86	1.21	1.13
Aqp4	aquaporin 4	1.58	-1.02	1.35	-1.04	-1.04
Oas2	2'-5'-oligoadenylate synthetase 2, 69/71kDa	1.58	1.80	2.00	-1.00	-1.05
Scarb1	scavenger receptor class B, member 1	1.57	1.11	1.71	1.19	1.04
Ebi3	Epstein-Barr virus induced 3	1.57	2.46	3.02	-1.04	-1.05
Ccr7	chemokine (C-C motif) receptor 7	1.56	3.77	1.57	2.49	1.39
Thy1	Thy-1 cell surface antigen	1.56	1.94	1.08	1.16	1.17
Stat4	signal transducer and activator of transcription 4	1.56	2.82	1.24	1.44	1.03
Ccr1	chemokine (C-C motif) receptor 1	1.56	2.19	2.00	1.06	1.01
Muc1	mucin 1, cell surface associated	1.56	1.30	3.27	-1.08	-1.08
Gzma	granzyme A (granzyme 1, cytotoxic T-lymphocyte-associated serine esterase 3)	1.56	3.39	1.32	1.50	1.18
Ccl7	chemokine (C-C motif) ligand 7	1.56	2.44	1.48	1.21	-1.18
Chi3l1	chitinase 3-like 1 (cartilage glycoprotein-39)	1.55	1.45	1.71	1.02	1.02
Il18bp	interleukin 18 binding protein	1.54	1.43	1.94	1.05	-1.15
Pim1	pim-1 oncogene	1.53	2.87	1.27	1.09	1.05
Abcg1	ATP-binding cassette, sub-family G (WHITE), member 1	1.53	1.26	1.53	1.17	1.06
Grn	granulin	1.52	1.49	1.71	-1.02	-1.11
Ctsz	cathepsin Z	1.52	1.88	1.36	-1.07	-1.16
Ticam1	toll-like receptor adaptor molecule 1	1.51	1.32	1.33	-1.01	1.19
Helz2	helicase with zinc finger 2, transcriptional coactivator	1.50	2.58	3.11	2.10	1.20
Lbp	lipopolysaccharide binding protein	1.49	1.29	-1.01	-1.22	-1.16
Grb7	growth factor receptor-bound protein 7	1.49	1.22	1.81	1.52	1.27
Abcg2	ATP-binding cassette, sub-family G (WHITE), member 2	1.48	1.23	1.46	1.05	1.17
Birc3	baculoviral IAP repeat containing 3	1.47	1.26	1.73	1.01	-1.05
Irf8	interferon regulatory factor 8	1.47	2.28	1.62	1.32	1.13
Nfkb2	nuclear factor of kappa light polypeptide gene enhancer in B-cells 2 (p49/p100)	1.47	2.67	1.88	1.02	1.04
Klf10	Kruppel-like factor 10	1.47	1.55	1.18	1.14	1.20
Cd86	CD86 molecule	1.46	2.14	1.26	1.08	-1.03
Casp1	caspase 1, apoptosis-related cysteine peptidase	1.45	2.52	1.93	1.05	-1.36
Guca2a	guanylate cyclase activator 2A (guanylin)	1.45	1.52	-1.85	-1.13	1.04
Hspa1a/ Hspa1b	heat shock 70kDa protein 1A	1.44	2.03	-1.05	1.13	1.33
Il3ra	interleukin 3 receptor, alpha (low affinity)	1.44	1.70	1.94	1.10	1.15
Hpse	heparanase	1.44	1.40	1.69	-1.22	-1.38
Abcb1	ATP-binding cassette, sub-family B (MDR/TAP), member 1	1.43	1.45	1.30	1.10	1.30
Junb	jun B proto-oncogene	1.43	1.82	1.81	1.51	1.07
Dnmt1	DNA (cytosine-5-)-methyltransferase 1	1.42	1.20	1.23	1.31	1.17
Vdr	vitamin D (1,25- dihydroxyvitamin D3) receptor	1.42	1.18	1.11	1.18	1.08
Serpinb9	serpin peptidase inhibitor, clade B (ovalbumin), member 9	1.42	2.18	1.29	1.01	1.03
Cd8b	CD8b molecule	1.42	2.28	1.35	1.12	-1.12
Cybb	cytochrome b-245, beta polypeptide	1.41	2.28	1.30	-1.04	-1.16
Erap1	endoplasmic reticulum aminopeptidase 1	1.40	1.88	1.94	1.13	1.08
Stat1	signal transducer and activator of transcription 1, 91kDa	1.40	2.33	2.62	1.17	1.01
Ccrl2	chemokine (C-C motif) receptor-like 2	1.39	2.17	1.23	-1.12	1.21
Csk	c-src tyrosine kinase	1.39	1.41	1.17	1.15	1.01
Usp18	ubiquitin specific peptidase 18	1.38	1.90	1.75	1.37	-1.04
Hmox1	heme oxygenase (decycling) 1	1.38	1.32	1.45	1.18	-1.04
F13a1	coagulation factor XIII, A1 polypeptide	1.37	1.57	2.22	-1.81	-2.44
Nfkbiz	nuclear factor of kappa light polypeptide gene enhancer in B-cells inhibitor, zeta	1.37	1.90	1.59	1.64	1.27
Tgif1	TGFB-induced factor homeobox 1	1.37	1.79	-1.02	1.12	1.06
Tnfrsf1a	tumor necrosis factor receptor superfamily, member 1A	1.37	1.53	1.21	-1.04	1.03
Ghrhr	growth hormone releasing hormone receptor	1.36	1.12	-1.72	1.11	-1.09
Mmp3	matrix metallopeptidase 3 (stromelysin 1, progelatinase)	1.36	1.95	2.32	1.08	-1.02

Symbol	Entrez Gene Name	*Asc*$^{-/-}$ 1	*Asc*$^{-/-}$ 2	*Nlrp3*$^{-/-}$ 1	*Il1b*$^{-/-}$ 1	C57BL/6
Tet2	tet methylcytosine dioxygenase 2	1.35	1.17	1.97	1.48	1.14
Olr1	oxidized low density lipoprotein (lectin-like) receptor 1	1.34	2.85	3.15	1.06	1.00
Ptpn6	protein tyrosine phosphatase, non-receptor type 6	1.33	1.13	1.56	1.01	1.11
Ly96	lymphocyte antigen 96	1.33	1.53	-1.04	1.06	1.02
Jun	jun proto-oncogene	1.33	1.81	-1.07	1.15	1.05
Gem	GTP binding protein overexpressed in skeletal muscle	1.32	1.47	-1.39	1.10	-1.04
Spi1	spleen focus forming virus (SFFV) proviral integration oncogene	1.31	1.56	2.34	1.19	-1.14
Il6	interleukin 6 (interferon, beta 2)	1.30	4.16	1.40	1.14	1.03
Cxcl2	chemokine (C-X-C motif) ligand 2	1.30	7.87	3.48	1.24	-1.00
Hspb1	heat shock 27kDa protein 1	1.30	1.23	-1.72	-1.08	1.05
Cry1	cryptochrome circadian clock 1	1.30	2.27	1.22	1.52	1.46
Adamts1	ADAM metallopeptidase with thrombospondin type 1 motif, 1	1.29	1.65	1.91	1.36	-1.09
Adrb2	adrenoceptor beta 2, surface	1.29	1.78	1.35	1.02	-1.06
Selp	selectin P (granule membrane protein 140kDa, antigen CD62)	1.28	1.83	1.13	-1.17	-1.17
Fas	Fas cell surface death receptor	1.27	2.09	1.23	1.10	-1.07
Cd163	CD163 molecule	1.27	1.71	1.80	-1.40	-2.56
B2m	beta-2-microglobulin	1.27	1.57	1.38	1.05	-1.02
Serpinb2	serpin peptidase inhibitor, clade B (ovalbumin), member 2	1.27	1.17	1.43	-1.30	-1.35
Mmp14	matrix metallopeptidase 14 (membrane-inserted)	1.26	1.42	1.41	-1.04	-1.04
Dusp5	dual specificity phosphatase 5	1.25	1.48	1.53	1.29	-1.14
Cysltr1	cysteinyl leukotriene receptor 1	1.25	1.49	1.33	-1.03	-1.16
Atf3	activating transcription factor 3	1.25	2.43	1.08	1.96	-1.90
Egr2	early growth response 2	1.24	2.28	1.59	1.14	-1.28
Traf1	TNF receptor-associated factor 1	1.24	2.08	1.47	1.22	1.13
Vasp	vasodilator-stimulated phosphoprotein	1.24	1.64	1.80	1.21	1.12
Dusp4	dual specificity phosphatase 4	1.23	1.87	2.08	1.17	-1.05
C1r	complement component 1, r subcomponent	1.23	1.35	1.23	1.04	-1.42
Nlrc5	NLR family, CARD domain containing 5	1.23	2.93	2.65	1.90	1.03
Aim2	absent in melanoma 2	1.23	1.53	2.72	1.33	1.00
Fcgr1a	Fc fragment of IgG, high affinity Ia, receptor (CD64)	1.23	1.55	1.39	1.06	-1.05
Cxcr3	chemokine (C-X-C motif) receptor 3	1.21	1.70	1.16	1.19	-1.06
Gfi1	growth factor independent 1 transcription repressor	1.21	1.39	1.52	1.15	1.09
Plat	plasminogen activator, tissue	1.21	1.56	1.57	-1.03	1.01
Ifit2	interferon-induced protein with tetratricopeptide repeats 2	1.20	3.06	1.42	1.20	-1.61
Ldha	lactate dehydrogenase A	1.20	1.54	1.44	-1.10	1.10
Fos	FBJ murine osteosarcoma viral oncogene homolog	1.19	2.23	1.71	1.28	-1.31
Ripk2	receptor-interacting serine-threonine kinase 2	1.19	1.55	1.15	-1.00	1.04
F2r	coagulation factor II (thrombin) receptor	1.17	1.46	1.10	1.17	1.22
Cd2	CD2 molecule	1.17	1.63	1.06	1.01	1.10
Itgb8	integrin, beta 8	1.17	2.11	1.29	1.00	1.29
Tnfsf10	tumor necrosis factor (ligand) superfamily, member 10	1.16	1.66	1.92	1.04	1.10
Cytip	cytohesin 1 interacting protein	1.16	1.55	1.69	1.26	1.03
Trem1	triggering receptor expressed on myeloid cells 1	1.16	1.56	1.48	-1.01	-1.08
Fat1	FAT atypical cadherin 1	1.15	1.26	2.07	1.38	1.06
Rc3h1	ring finger and CCCH-type domains 1	1.15	1.16	1.57	1.32	1.27
Mapkapk2	mitogen-activated protein kinase-activated protein kinase 2	1.14	1.35	1.46	1.27	-1.04
Zfp36	ZFP36 ring finger protein	1.13	1.79	2.14	1.22	-1.02
Cort	cortistatin	1.13	1.06	-1.66	-1.13	1.05
Sell	selectin L	1.12	1.97	1.56	-1.02	-1.07
Map4k1	mitogen-activated protein kinase kinase kinase kinase 1	1.12	1.78	1.41	-1.09	-1.08
Ccl2	chemokine (C-C motif) ligand 2	1.12	2.22	1.20	1.09	-1.09
Igf1	insulin-like growth factor 1 (somatomedin C)	1.11	1.37	1.03	-1.93	-1.49
Arf6	ADP-ribosylation factor 6	1.11	1.41	-1.42	1.10	1.04
Trim16	tripartite motif containing 16	1.10	1.10	1.44	1.18	-1.04
Ifnar1	interferon (alpha, beta and omega) receptor 1	1.10	1.57	1.02	-1.03	-1.05
Hla-E	major histocompatibility complex, class I, E	1.10	1.57	1.11	1.15	1.03
Rel	v-rel avian reticuloendotheliosis viral oncogene homolog	1.10	2.09	1.34	-1.18	1.06
Tlr3	toll-like receptor 3	1.10	1.18	1.24	1.47	-1.02
Nlrp3	NLR family, pyrin domain containing 3	1.09	1.28	1.52	1.13	-1.03
Ccl22	chemokine (C-C motif) ligand 22	1.08	3.75	1.04	1.50	1.50
Ets1	v-ets avian erythroblastosis virus E26 oncogene homolog 1	1.07	1.56	1.13	-1.07	1.01
Srf	serum response factor (c-fos serum response element-binding transcription factor)	1.07	1.19	1.50	1.09	-1.10
Cish	cytokine inducible SH2-containing protein	1.06	1.59	1.31	-1.07	-1.05
Vip	vasoactive intestinal peptide	1.06	-1.16	-1.26	-1.56	-1.32
C6	complement component 6	1.05	1.48	1.29	-1.11	-1.01
Hmga1	high mobility group AT-hook 1	1.05	1.46	1.27	1.11	1.18
Bax	BCL2-associated X protein	1.04	1.21	-1.47	1.13	1.09
Bgn	biglycan	1.04	-1.15	-1.54	-1.32	-1.38
Mmp12	matrix metallopeptidase 12 (macrophage elastase)	1.02	-1.48	-1.06	-1.21	1.20

Symbol	Entrez Gene Name	*Asc*$^{-/-}$ 1	*Asc*$^{-/-}$ 2	*Nlrp3*$^{-/-}$ 1	*Il1b*$^{-/-}$ 1	C57BL/6
Ifng	interferon, gamma	1.02	2.32	1.21	1.15	-1.03
Cdkn1a	cyclin-dependent kinase inhibitor 1A (p21, Cip1)	1.02	1.22	-1.15	1.51	1.33
Syvn1	synovial apoptosis inhibitor 1, synoviolin	1.01	1.14	1.56	1.22	1.05
Sugt1	SGT1, suppressor of G2 allele of SKP1 (S. cerevisiae)	1.01	1.17	-1.70	-1.04	-1.01
Cd40	CD40 molecule, TNF receptor superfamily member 5	1.01	2.11	1.13	-1.15	-1.01
Alox15	arachidonate 15-lipoxygenase	-1.01	1.24	-1.49	-1.42	-1.65
Il22	interleukin 22	-1.02	1.93	1.22	1.15	1.01
Apbb1	amyloid beta (A4) precursor protein-binding, family B, member 1 (Fe65)	-1.03	-1.15	-1.85	-1.26	-1.02
Aimp1	aminoacyl tRNA synthetase complex-interacting multifunctional protein 1	-1.03	-1.07	-1.56	1.02	-1.08
Mmp2	matrix metallopeptidase 2 (gelatinase A, 72kDa gelatinase, 72kDa type IV collagenase)	-1.03	-1.29	-1.30	-1.47	-1.01
Il1r2	interleukin 1 receptor, type II	-1.04	1.47	2.10	1.10	-1.33
Hspg2	heparan sulfate proteoglycan 2	-1.04	-1.14	-1.42	-1.22	1.13
Fst	follistatin	-1.04	1.59	1.36	-1.15	-1.33
Mapk1	mitogen-activated protein kinase 1	-1.05	-1.07	-1.44	1.12	1.08
Ccl4	chemokine (C-C motif) ligand 4	-1.05	2.91	1.19	1.07	1.08
Plau	plasminogen activator, urokinase	-1.05	-1.35	-1.42	-1.03	-1.08
Map2k3	mitogen-activated protein kinase kinase 3	-1.06	-1.00	-1.59	-1.10	1.01
Nos3	nitric oxide synthase 3 (endothelial cell)	-1.06	1.95	2.65	-1.10	1.19
Ppp1r12a	protein phosphatase 1, regulatory subunit 12A	-1.06	-1.51	-1.46	-1.05	-1.00
Hsd11b1	hydroxysteroid (11-beta) dehydrogenase 1	-1.06	-1.18	-1.51	-1.39	-1.20
Gbp2	guanylate binding protein 2, interferon-inducible	-1.07	1.60	1.73	1.03	-1.10
Ptgir	prostaglandin I2 (prostacyclin) receptor (IP)	-1.07	1.25	1.55	-1.05	1.04
Ccr4	chemokine (C-C motif) receptor 4	-1.07	-1.03	-1.44	-1.04	1.06
Kcnh2	potassium voltage-gated channel, subfamily H (eag-related), member 2	-1.07	-1.23	-2.57	-1.66	1.02
Fgf7	fibroblast growth factor 7	-1.08	-1.14	-1.46	-1.05	1.02
Nr1i3	nuclear receptor subfamily 1, group I, member 3	-1.09	1.03	-1.47	1.05	-1.07
Tnfaip6	tumor necrosis factor, alpha-induced protein 6	-1.09	1.53	1.52	-1.01	-1.04
Rasa2	RAS p21 protein activator 2	-1.09	-1.07	1.42	1.05	-1.02
Ampd3	adenosine monophosphate deaminase 3	-1.09	1.07	-1.24	-1.16	-1.41
Peli2	pellino E3 ubiquitin protein ligase family member 2	-1.10	-1.04	-1.42	-1.12	-1.00
Ptgis	prostaglandin I2 (prostacyclin) synthase	-1.11	-1.34	-2.16	-1.34	-1.88
Notch1	notch 1	-1.12	-1.41	-1.07	1.19	1.08
Pla2g2d	phospholipase A2, group IID	-1.13	-1.08	-1.47	-1.07	-1.08
Ptgs1	prostaglandin-endoperoxide synthase 1 (prostaglandin G/H synthase and cyclooxygenase)	-1.13	-1.19	-1.50	1.02	-1.09
Trh	thyrotropin-releasing hormone	-1.14	-1.35	-1.52	1.00	-1.06
Osm	oncostatin M	-1.14	1.85	1.37	-1.10	1.09
Tbx21	T-box 21	-1.14	1.42	1.05	1.21	1.33
Pin1	peptidylprolyl cis/trans isomerase, NIMA-interacting 1	-1.15	-1.02	-1.83	-1.02	1.03
Slc22a4	solute carrier family 22 (organic cation/zwitterion transporter), member 4	-1.16	-1.10	-1.21	-1.17	-1.47
Mmp11	matrix metallopeptidase 11 (stromelysin 3)	-1.16	-1.39	-1.55	-1.23	-1.24
Phlda1	pleckstrin homology-like domain, family A, member 1	-1.16	1.24	1.50	-1.05	-1.10
Npy	neuropeptide Y	-1.17	-1.15	-1.04	-1.52	-1.18
Agt	angiotensinogen (serpin peptidase inhibitor, clade A, member 8)	-1.17	-1.12	1.82	-1.13	-1.05
Clec11a	C-type lectin domain family 11, member A	-1.18	-1.44	-1.62	-1.62	-1.12
Fosb	FBJ murine osteosarcoma viral oncogene homolog B	-1.18	-1.07	-1.30	1.03	-1.43
Anxa9	annexin A9	-1.20	-1.42	-1.31	-1.29	-1.02
Igfbp6	insulin-like growth factor binding protein 6	-1.21	1.15	-2.17	-1.86	-2.80
Gadd45a	growth arrest and DNA-damage-inducible, alpha	-1.22	-1.11	-1.55	1.31	1.01
Ptprn	protein tyrosine phosphatase, receptor type, N	-1.22	-1.43	-1.22	-1.07	-1.00
Id3	inhibitor of DNA binding 3, dominant negative helix-loop-helix protein	-1.25	1.24	-1.69	1.00	1.36
Plk2	polo-like kinase 2	-1.25	-1.18	-1.59	-1.09	-1.00
Smpd2	sphingomyelin phosphodiesterase 2, neutral membrane (neutral sphingomyelinase)	-1.27	-2.01	-2.00	-1.34	-1.08
Cebpa	CCAAT/enhancer binding protein (C/EBP), alpha	-1.27	1.12	1.52	-1.00	-1.17
Pdcd4	programmed cell death 4 (neoplastic transformation inhibitor)	-1.28	-1.45	-1.99	-1.02	-1.05
Nfe2l2	nuclear factor, erythroid 2-like 2	-1.29	-1.17	-1.54	1.10	1.08
Cdkn1c	cyclin-dependent kinase inhibitor 1C (p57, Kip2)	-1.30	-1.03	-1.64	-1.67	-1.27
Tfam	transcription factor A, mitochondrial	-1.30	-1.23	-1.72	1.04	1.03
Pgf	placental growth factor	-1.32	-1.28	-1.48	1.05	-1.48
Sirt1	sirtuin 1	-1.33	-1.15	-1.29	1.68	-1.06
Bag4	BCL2-associated athanogene 4	-1.36	-1.29	-1.57	1.00	-1.04
Txnip	thioredoxin interacting protein	-1.37	-1.59	-1.99	-1.18	-1.10
Penk	proenkephalin	-1.38	1.55	2.15	-1.27	-1.34

Symbol	Entrez Gene Name	*Asc*$^{-/-}$ 1	*Asc*$^{-/-}$ 2	*Nlrp3*$^{-/-}$ 1	*Il1b*$^{-/-}$ 1	C57BL/6
Nr4a1	nuclear receptor subfamily 4, group A, member 1	-1.38	1.39	-1.60	1.81	-1.24
Rarb	retinoic acid receptor, beta	-1.39	-1.63	-2.59	-1.26	-1.04
Thbd	thrombomodulin	-1.39	1.15	1.12	-1.45	-1.46
Cma1	chymase 1, mast cell	-1.40	1.70	1.01	-1.08	-1.55
Dusp1	dual specificity phosphatase 1	-1.41	1.31	1.19	-1.18	-1.49
Ccrn4l	CCR4 carbon catabolite repression 4-like (S. cerevisiae)	-1.41	-2.19	1.91	-1.01	1.16
Agtr1	angiotensin II receptor, type 1	-1.42	-1.23	-1.43	-1.69	-1.31
Ambp	alpha-1-microglobulin/bikunin precursor	-1.43	1.03	-1.06	-1.16	-1.08
Klf2	Kruppel-like factor 2	-1.43	1.27	1.24	1.40	-1.37
Acan	aggrecan	-1.43	-1.24	-1.39	-1.13	-1.16
Myh11	myosin, heavy chain 11, smooth muscle	-1.44	-2.01	-2.13	-1.03	-1.23
Alb	albumin	-1.44	-1.26	-1.19	-1.10	-1.04
Lrpap1	low density lipoprotein receptor-related protein associated protein 1	-1.45	-1.09	-1.19	-1.23	-1.24
Enpp1	ectonucleotide pyrophosphatase/phosphodiesterase 1	-1.45	-1.15	-1.26	1.09	1.01
Twist1	twist family bHLH transcription factor 1	-1.46	1.00	-1.05	-1.24	-1.47
Fam129a	family with sequence similarity 129, member A	-1.46	-1.74	-3.09	-1.45	-1.33
Pthlh	parathyroid hormone-like hormone	-1.46	-1.32	-1.44	-1.01	-1.28
Vegfc	vascular endothelial growth factor C	-1.48	-1.12	-1.58	-1.25	1.05
Scnn1b	sodium channel, non-voltage-gated 1, beta subunit	-1.50	-1.65	-1.92	-1.02	1.02
Smad2	SMAD family member 2	-1.50	-1.49	-1.26	1.09	1.06
Isg20	interferon stimulated exonuclease gene 20kDa	-1.50	-1.07	-2.53	-1.25	-1.20
Il15	interleukin 15	-1.50	1.07	-1.23	-1.26	-1.15
Cdh22	cadherin 22, type 2	-1.50	-1.32	1.24	1.24	1.18
Timp3	TIMP metallopeptidase inhibitor 3	-1.50	-1.64	-2.41	-1.35	-1.52
Oxtr	oxytocin receptor	-1.54	-1.29	-1.06	-1.04	-1.02
Pde5a	phosphodiesterase 5A, cGMP-specific	-1.55	-2.19	-1.66	-1.13	-1.02
Tgfb3	transforming growth factor, beta 3	-1.58	-2.32	-2.45	1.01	-1.90
Pla2g4a	phospholipase A2, group IVA (cytosolic, calcium-dependent)	-1.59	-1.45	-1.52	1.12	-1.10
Srebf1	sterol regulatory element binding transcription factor 1	-1.61	-1.11	-1.22	-1.02	1.20
Mylk	myosin light chain kinase	-1.62	-2.70	-1.88	-1.17	-1.19
Cdkn2a	cyclin-dependent kinase inhibitor 2A	-1.63	-1.20	-1.01	1.12	1.22
Pparg	peroxisome proliferator-activated receptor gamma	-1.63	-1.40	-2.39	1.11	-1.14
Isl1	ISL LIM homeobox 1	-1.63	-1.10	-1.02	-1.19	-1.02
Stmn2	stathmin-like 2	-1.64	-1.54	-2.30	-1.40	-1.53
Prnp	prion protein	-1.65	-1.12	-1.61	-1.32	-1.05
Ppara	peroxisome proliferator-activated receptor alpha	-1.65	-1.93	-2.34	1.01	-1.05
Irak2	interleukin-1 receptor-associated kinase 2	-1.67	-1.43	-1.70	-1.05	-1.07
F3	coagulation factor III (thromboplastin, tissue factor)	-1.68	-1.27	1.32	-1.36	-1.98
G6pd	glucose-6-phosphate dehydrogenase	-1.69	-1.26	-1.70	1.06	1.06
Popdc2	popeye domain containing 2	-1.71	-2.05	-1.80	-1.09	-1.07
Avpr1a	arginine vasopressin receptor 1A	-1.72	-1.74	-2.66	1.02	1.14
Hes1	hes family bHLH transcription factor 1	-1.73	-1.66	-1.21	1.27	1.10
Abcc3	ATP-binding cassette, sub-family C (CFTR/MRP), member 3	-1.74	-1.34	-1.89	1.09	1.25
Lum	lumican	-1.74	-1.32	-2.58	-2.73	-1.42
Ccl24	chemokine (C-C motif) ligand 24	-1.75	-1.59	-1.57	-1.70	-1.47
Csrnp1	cysteine-serine-rich nuclear protein 1	-1.76	1.02	-1.11	1.04	1.07
Pim3	pim-3 oncogene	-1.76	-1.20	-1.49	1.01	-1.18
Fabp4	fatty acid binding protein 4, adipocyte	-1.79	-1.05	1.01	-1.05	-1.11
Apol6	apolipoprotein L, 6	-1.79	1.01	1.64	-1.09	1.07
Nr3c2	nuclear receptor subfamily 3, group C, member 2	-1.83	-2.07	-2.84	1.01	-1.11
Cat	catalase	-1.88	-1.05	1.03	-1.01	-1.16
Thbs1	thrombospondin 1	-1.89	-1.50	-1.10	1.41	-1.89
Ppp1r14a	protein phosphatase 1, regulatory (inhibitor) subunit 14A	-1.91	-4.92	-4.24	-1.83	-1.87
Serpine1	serpin peptidase inhibitor, clade E (nexin, plasminogen activator inhibitor type 1), member 1	-1.95	-1.54	-1.79	1.29	-1.64
Nr1d1	nuclear receptor subfamily 1, group D, member 1	-1.99	-1.93	-1.97	-1.17	-2.10
Myc	v-myc avian myelocytomatosis viral oncogene homolog	-2.00	-1.58	-2.56	1.17	-1.11
Sele	selectin E	-2.00	-2.83	-1.87	1.31	1.05
Cry2	cryptochrome circadian clock 2	-2.02	-1.39	-1.50	1.05	-1.21
Crh	corticotropin releasing hormone	-2.04	-2.17	1.26	1.04	1.04
Ccl11	chemokine (C-C motif) ligand 11	-2.14	-1.06	-1.50	-1.39	-3.10
Lep	leptin	-2.17	-1.23	-1.01	1.46	2.76
Cyp2e1	cytochrome P450, family 2, subfamily E, polypeptide 1	-2.23	-1.07	2.84	-1.24	-2.12
G0s2	G0/G1switch 2	-2.23	1.68	3.05	-1.20	2.05
Bmp7	bone morphogenetic protein 7	-2.23	-2.13	-1.85	1.06	-1.01
Ugdh	UDP-glucose 6-dehydrogenase	-2.25	-2.06	-4.31	1.01	1.08
Scnn1g	sodium channel, non-voltage-gated 1, gamma subunit	-2.29	-2.51	-3.07	1.22	1.14
Irs1	insulin receptor substrate 1	-2.36	-1.80	-1.43	-1.29	-1.36
Ccnd1	cyclin D1	-2.48	-1.89	-2.76	-1.13	-1.44
Scd	stearoyl-CoA desaturase (delta-9-desaturase)	-2.52	1.01	2.45	-1.10	-1.13
Gclc	glutamate-cysteine ligase, catalytic subunit	-2.58	-2.21	-2.30	1.01	1.01

Symbol	Entrez Gene Name	*Asc*$^{-/-}$ 1	*Asc*$^{-/-}$ 2	*Nlrp3*$^{-/-}$ 1	*Il1b*$^{-/-}$ 1	C57BL/6
Slc14a1	solute carrier family 14 (urea transporter), member 1 (Kidd blood group)	-2.61	-1.99	-1.72	1.05	1.03
Bmp4	bone morphogenetic protein 4	-2.61	-3.88	-4.79	-1.67	-1.60
Mt1h	metallothionein 1H	-2.62	-3.62	-3.46	-1.18	-1.91
Adipoq	adiponectin, C1Q and collagen domain containing	-2.62	-1.14	4.12	-1.30	-1.22
Pla2g7	phospholipase A2, group VII (platelet-activating factor acetylhydrolase, plasma)	-2.65	-2.99	-2.67	-1.19	-1.26
Cd36	CD36 molecule (thrombospondin receptor)	-3.06	-1.13	1.67	-1.30	-1.03
Lpl	lipoprotein lipase	-3.06	1.05	2.41	-1.07	1.10
Irs2	insulin receptor substrate 2	-3.28	-2.68	-3.28	1.04	1.09
Nqo1	NAD(P)H dehydrogenase, quinone 1	-3.52	-3.78	-9.11	1.05	-1.39
Alpl	alkaline phosphatase, liver/bone/kidney	-3.67	-2.52	-4.58	-1.16	1.22
Bmp2	bone morphogenetic protein 2	-5.02	-3.38	-4.15	1.03	-1.58
Dbp	D site of albumin promoter (albumin D-box) binding protein	-6.41	-14.22	-15.29	-1.61	-7.75
Cyp1a1	cytochrome P450, family 1, subfamily A, polypeptide 1	-9.55	-16.74	-6.79	1.12	-3.74
Scube2	signal peptide, CUB domain, EGF-like 2	-15.38	-11.43	-20.27	1.20	1.16
