# Supplementary material for: Molecular Basis of Acute Cystitis Reveals Susceptibility Genes and Immunotherapeutic Targets
Source: PLoS Pathog. 2016 Oct 12;12(10):e1005848. doi: 10.1371/journal.ppat.1005848 (PMC5061333; doi:10.1371/journal.ppat.1005848)
Supplement: S3 Table — (PDF) [file ppat.1005848.s013.pdf]

S3 Table. Primers used to amplify the *MMP7* promoter and promoter flanks.

Fragment		Primers sequence (5'-3')	Amplicon lenght
**P1**	for	ATTTATATAGCTTCTCAGCCTCGAATGT	259
	rev	TGATACCTATGAGAGCAGTCATTTGAC	
**P2**	for	GCTCTCATAGGTATCATTCAGGACATTAT	252
	rev	TGTATGGAGAACCACAGGATTTTGG	
**P3**	for	AAATAGAACATCACCAAAATCCTGTGG	267
	rev	AGATTTTTGTTGGCTTGGTATTTTTTTGT	
**P4**	for	ACAAAAAAATACCAAGCCAACAAAAATC	276
	rev	TTGGTAAGAATGGAGTCATTGGG	
**P5**	for	ATTCTTACCAAAAATGAAAGTGAGGCC	196
	rev	CCCATGATGTATTAGAGTCAAAAGCC	
**F1**	for	ATTTGAAGACAGAATAATTACATAGTCACAGAGA	274
	rev	CACAGGGTCTATGATAACACAGAGAAT	
**F2**	for	TGAGGTCAACCAGCCCTATCT	212
	rev	AAGTCAAGTCCAGTTCTGCTACTAAT	
**F3**	for	TAGTAGCAGAACTGGACTTGACTTTC	205
	rev	ATTCAGATACTGAGAAACTTTAGAGAAATTCC	
**F4**	for	TTCCTGGGAATTTCTCTAAAGTTTCTCA	258
	rev	GCAATTTATTTCACTTCTCTGAGCTGT	
**F5**	for	GGGTTATATGATGCCTGATAATATTGTTGT	124
	rev	AATTGCATATTCCTGGGCGGT	
